# *In vivo* small animal micro-CT using nanoparticle contrast agents

**DOI:** 10.3389/fphar.2015.00256

**Published:** 2015-11-04

**Authors:** Jeffrey R. Ashton, Jennifer L. West, Cristian T. Badea

**Affiliations:** ^1^Department of Biomedical Engineering, Duke University, DurhamNC, USA; ^2^Department of Radiology, Center for In Vivo Microscopy, Duke University Medical Center, DurhamNC, USA

**Keywords:** micro-CT, small animal imaging, nanoparticles, contrast agents, spectral imaging

## Abstract

Computed tomography (CT) is one of the most valuable modalities for *in vivo* imaging because it is fast, high-resolution, cost-effective, and non-invasive. Moreover, CT is heavily used not only in the clinic (for both diagnostics and treatment planning) but also in preclinical research as micro-CT. Although CT is inherently effective for lung and bone imaging, soft tissue imaging requires the use of contrast agents. For small animal micro-CT, nanoparticle contrast agents are used in order to avoid rapid renal clearance. A variety of nanoparticles have been used for micro-CT imaging, but the majority of research has focused on the use of iodine-containing nanoparticles and gold nanoparticles. Both nanoparticle types can act as highly effective blood pool contrast agents or can be targeted using a wide variety of targeting mechanisms. CT imaging can be further enhanced by adding spectral capabilities to separate multiple co-injected nanoparticles *in vivo*. Spectral CT, using both energy-integrating and energy-resolving detectors, has been used with multiple contrast agents to enable functional and molecular imaging. This review focuses on new developments for *in vivo* small animal micro-CT using novel nanoparticle probes applied in preclinical research.

## Introduction

X-ray computed tomography (CT) is one of the most powerful and widely used imaging modalities in modern clinical practice. CT provides non-invasive three-dimensional imaging capabilities at lower cost and higher spatial and temporal resolution than other imaging modalities such as MRI and PET (Kircher and Willmann, 2012). CT imaging can reveal a patient’s anatomy in exquisite detail and is extremely useful in the diagnosis of a wide variety of diseases. CT systems with high resolution (also known as micro-CT systems) have been developed over the last few decades and have been used with great success in small animal studies. With micro-CT, animals can be non-invasively imaged *in vivo* multiple times over the course of a preclinical study, which significantly decreases the number of animals required compared to methods requiring *ex vivo* analysis. Additionally, the continued development of micro-CT can help to test and optimize imaging advances for translation to clinical CT. This review provides an overview of micro-CT imaging principles and applications of micro-CT in preclinical small animal studies, with a special emphasis on the use of nanoparticle contrast agents and spectral imaging methods that could serve well in drug discovery and pharmacological research.

## Micro-Ct Imaging Principles

### Imaging System

**FIGURE 1:**
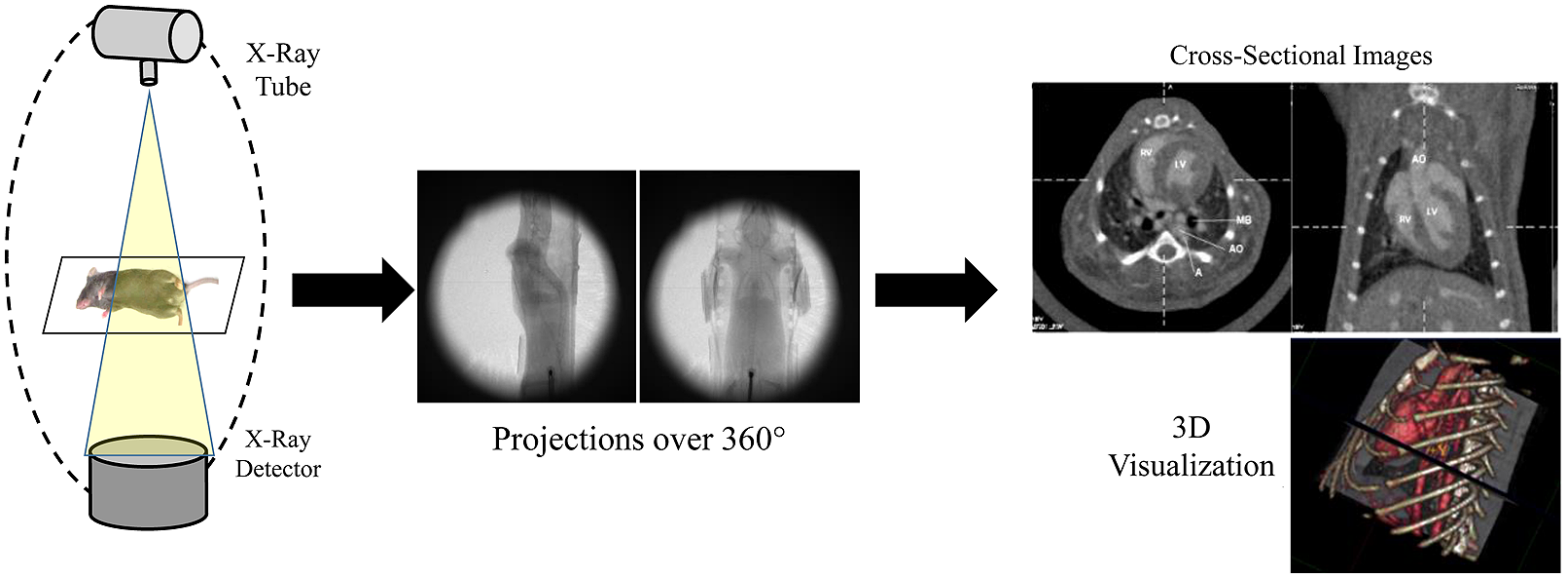
**Schematic of a micro-CT system.** Multiple x-ray projections are acquired over a 360° rotation around the subject. These individual x-ray projections are then reconstructed to produce 2D cross-sectional images and 3D volumes.

A CT system consists of an x-ray source and x-ray detectors, between which the subject is placed. In clinical CT, the x-ray source and detectors rotate around the subject to produce projections of x-ray attenuation through the body at many different angles. For micro-CT, the x-ray source and detectors may also be static, while the small animal is rotated between them. The x-ray projections acquired at each angle of rotation are then used to reconstruct tomographic images, which are visualized as 2D slices or 3D volumes of the specimen. The most common reconstruction method for micro-CT is a cone-beam implementation of filtered back projection (Feldkamp et al., 1984). A schematic of a micro-CT system and reconstruction process is shown in **Figure 1.**

### X-ray Generation

**FIGURE 2:**
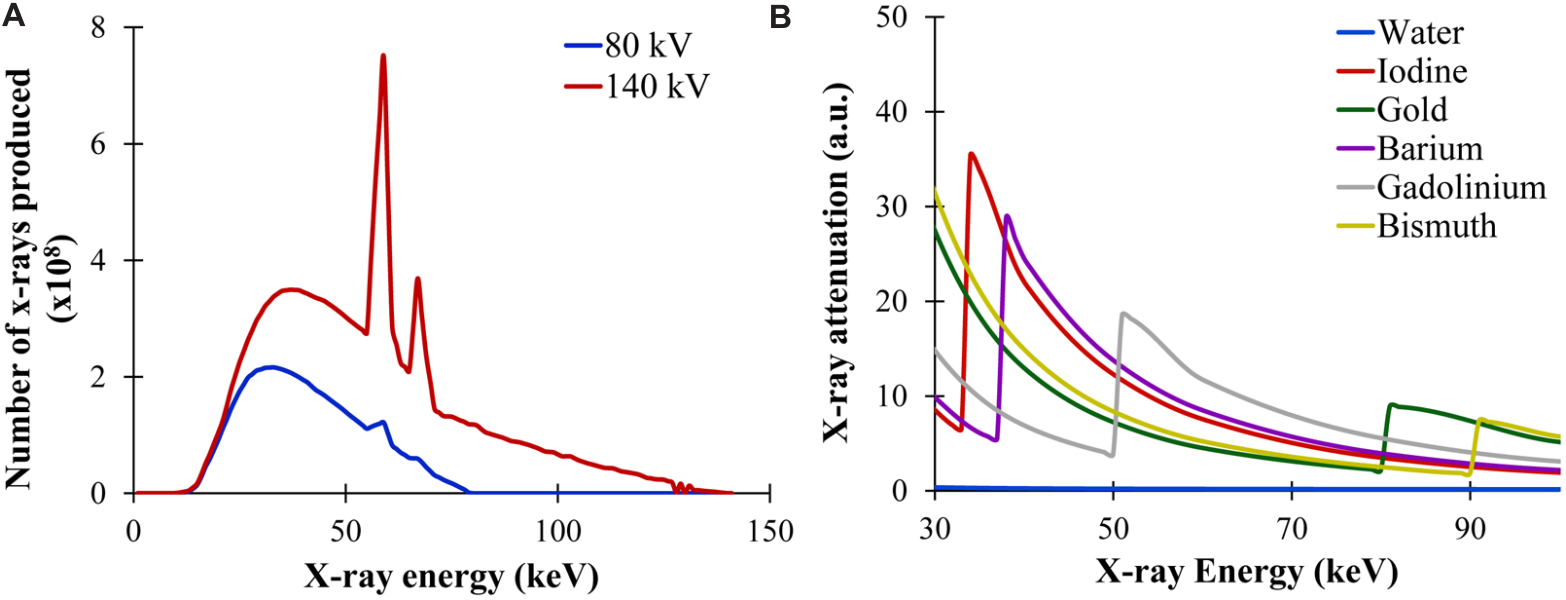
**X-ray production and attenuation.** **(A)** X-ray energy spectra produced at two different tube voltages: 80 and 140 kV. Both the number of photons produced and the mean energy of the spectrum increases with higher voltage. **(B)** X-ray attenuation as a function of x-ray energy for multiple materials. In general, the x-ray attenuation rapidly drops with increasing x-ray energy. At the K-edge of each material, there is a sharp rise in attenuation due to the photoelectric absorption at that energy.

X-rays are generated by accelerating electrons across a high voltage to collide with an anode composed of a high atomic number, high melting point material (commonly tungsten). Interactions between the electrons and the tungsten anode lead to the production of x-rays with a broad energy spectrum. The maximum energy of the x-ray spectrum is determined by the voltage applied in the x-ray tube. As tube voltage increases, the mean x-ray energy and number of photons produced both increase. This is demonstrated in **Figure 2A** for a tungsten anode operating at two different voltages: 80 and 140 kV. The energy of the produced x-rays is an important determinant of their absorption by a given material. This energy spectrum can be modified by filtration through metal filters. Filtration is primarily used to increase the mean energy of the x-ray spectrum by removing low energy photons. Filtration can be used to both reduce radiation dose and improve image quality, and filtration can be optimized depending on the imaging task (Hupfer et al., 2012). Micro-CT x-ray tubes differ from clinical x-ray tubes in that they usually have a much smaller focal spot (area where the electron beam interacts with the anode), which reduces the source function blur (i.e., penumbra blurring) and thereby greatly improves the maximum image resolution. This increased resolution is necessary for imaging small animals which have much smaller features than humans.

### X-ray Attenuation

X-rays travel from the focal point of the x-ray tube, through the subject, and on to the x-ray detector. The x-ray detector measures the relative amount of x-rays absorbed by the subject at any given position. X-ray attenuation is given by

$$I=I_{0}e^{-\mu x}$$

where I is the intensity of the x-rays transmitted through the subject, I_0_ is the original intensity of the x-rays incident on the object, μ is the linear attenuation coefficient of the object, and x is the thickness of the object. Therefore, absorption of x-rays by a material is dependent on the thickness of the material and on the material-dependent attenuation coefficient. Diagnostic x-rays can be absorbed by a material via two primary mechanisms: compton scattering and the photoelectric effect.

Compton scattering occurs when an x-ray photon collides with an outer shell electron within the subject. Upon collision, the electron absorbs a portion of the x-ray energy and is ejected from the atom. The x-ray photon is deflected from its original direction and loses some energy. This scattering can occur in all directions and can lead to noise at the detector. The amount of Compton scattering that occurs within an object depends primarily on the energy of the incident x-ray photon and the density of the object. Compton scattering decreases slightly with increasing photon energy, so higher energy x-rays are better able to pass through a patient without attenuation. The density of outer shell electrons increases with the mass density of a material, so denser materials tend to have more Compton scattering and therefore more x-ray attenuation.

The photoelectric effect occurs when an x-ray photon transfers all of its energy to an inner shell electron within the subject. This electron is ejected from the atom and its vacancy is subsequently filled by an outer-shell electron, which leads to the release of a secondary photon. The photoelectric effect is highly dependent on both the energy of the incident x-ray and the atomic weight of the object. The photoelectric effect is strongest when the x-ray energy matches the binding energy of the inner-shell electrons. As x-ray energy increases, the likelihood of the photoelectric effect drops rapidly, proportional to the inverse cube of the x-ray energy (1/E^3^). If the x-ray energy is below the energy of a particular electron shell, then none of those electrons can participate in the photoelectric effect because the x-ray does not have enough energy to overcome the electron binding energy. This leads to the K-edge effect, where the probability of absorption due to the photoelectric effect jumps abruptly as the x-ray energy increases above the K-shell electron binding energy. The photoelectric effect is also proportional to the cube of a material’s atomic number (Z^3^), so high atomic weight materials exhibit a much stronger photoelectric effect than low atomic weight materials. This is why contrast agents for CT traditionally include high atomic weight elements (e.g., iodine, barium). The K-edge effect is shown in **Figure 2B**, which demonstrates the relative probability of x-ray photon attenuation at different x-ray energies for several high Z materials such as iodine, gold, barium, gadolinium, bismuth.

## Applications Of Non-Contrast-Enhanced Micro-Ct

Micro-CT images only demonstrate high contrast when there are large differences between material densities (Compton scattering) or atomic weights (photoelectric effect) within the patient. In the case of soft tissue imaging, there is very little natural contrast so an exogenous high atomic weight contrast agent must be administered for effective imaging (Yu and Watson, 1999). However, non-contrast-enhanced micro-CT performs well for bone and lung imaging, both of which have high inherent contrast in the absence of exogenous contrast agents.

### Bone Imaging

**FIGURE 3:**
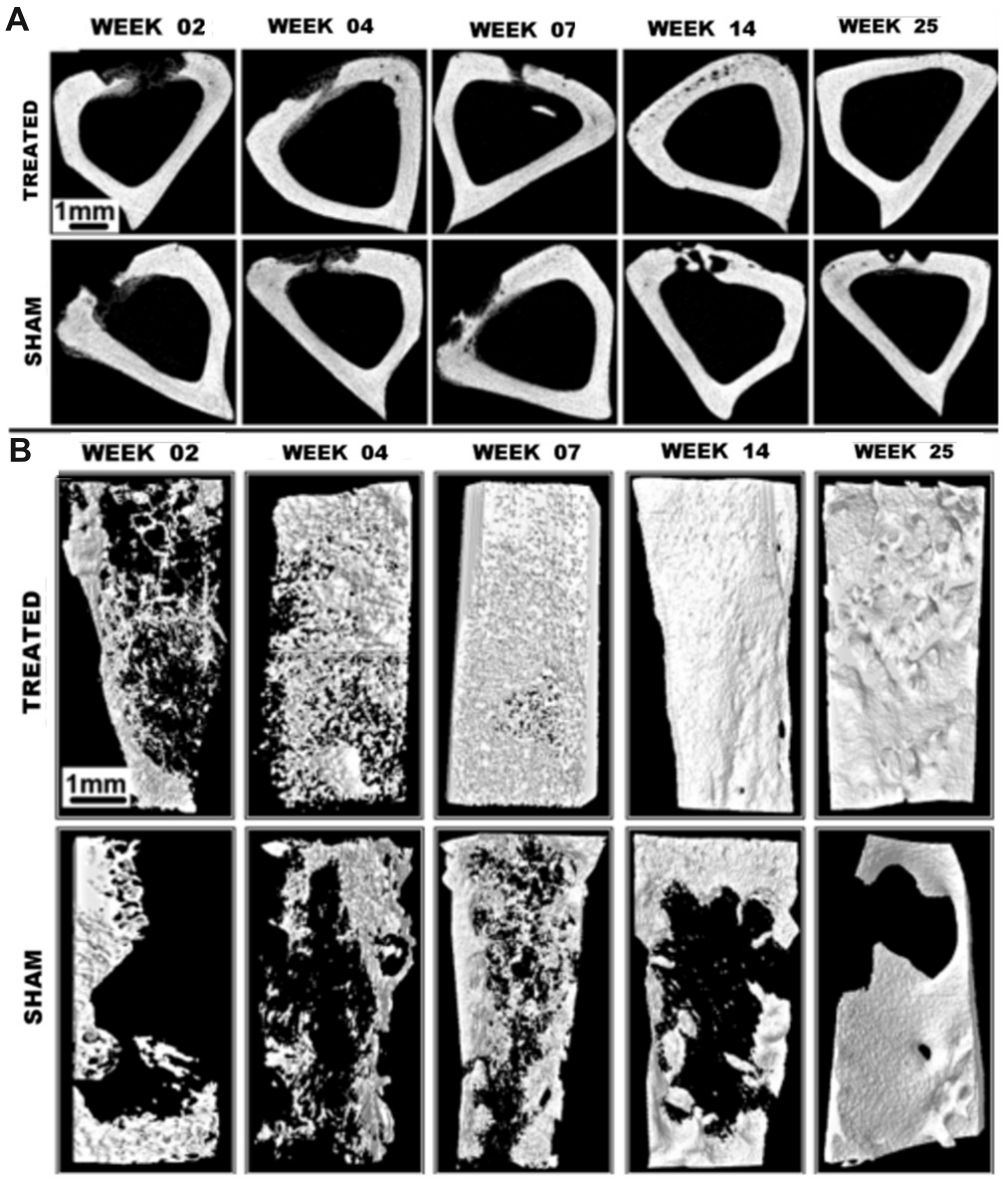
**Tibial bone defect micro-CT imaging.** **(A)** Axial micro-CT cross-sections and **(B)** 3D reconstructions of tibial bone defects after treatment with an osteoinductive gel scaffold. Longitudinal imaging was performed up to 25 weeks. Addition of the treatment gel significantly improves healing of the bone defect. Reprinted from (Sagar et al., 2013) under the Creative Commons Attribution License.

Micro-CT is well-suited for bone imaging because of the natural contrast between bone and soft tissues, which is due to the high effective atomic weight of bone. This makes micro-CT extremely valuable for non-invasive, high-resolution bone imaging without the need of an exogenous contrast agent. Bone imaging was one of the very first common applications of micro-CT for small animal imaging (Feldkamp et al., 1989; Kinney et al., 1995). Micro-CT can accurately quantify a variety of bone parameters, including cross-sectional area, cortical thickness, bone mineral density, bone volume, bone surface ratio, and trabecular thickness (Bouxsein et al., 2010). Structural micro-CT studies have examined bone architecture (Waarsing et al., 2005; Hsu et al., 2014), bone remodeling (David et al., 2003; Cowan et al., 2007), and osteoarthritis (Appleton et al., 2007; McErlain et al., 2008). Micro-CT has also been used to monitor bone healing after treatment with basic fibroblast growth factor (Yao et al., 2005), vascular endothelial growth factor gene therapy (Li et al., 2009), or stem cell therapy (Lee et al., 2009). Micro-CT can also be used to longitudinally track bone loss and structural changes following radiation therapy and bone marrow transplantation (Dumas et al., 2009) or after spinal cord injury (Jiang et al., 2006). In the case of osteoporosis, micro-CT measurements have been used to study disease progression after ovariectomy (Laib et al., 2001) or immobilization (Laib et al., 2000). Micro-CT has also been used to study early bone development and growth (Guldberg et al., 2004). Additionally, micro-CT has been used extensively in studies of bone regeneration (Umoh et al., 2009) and bone tissue engineering (Lin et al., 2005; Ho and Hutmacher, 2006). In these cases, micro-CT can quantify mineral content, porosity, and connectivity, as well as accurately determine three-dimensional structures. **Figure 3** illustrates the use of micro-CT to evaluate healing of a tibial bone defect after treatment with an osteoinductive gel scaffold (Sagar et al., 2013). This study shows the ability of micro-CT to produce both 2D cross-sectional bone images as well 3D reconstructions of entire bones. Within the 3D reconstructions, bone microarchitecture is clearly visualized.

### Lung Imaging

The large difference in density between air-filled lungs and soft tissues creates high contrast for lung imaging, which makes CT an extremely useful modality for studying the lung. The primary difficulty in imaging the lungs is respiratory motion. Small animal respiratory rates are 3–4 times the average human respiratory rate, so completing an entire scan between breaths is not practical. Instead, various gating strategies are used which allow researchers to acquire each projection at the same stage in the respiratory cycle, so that there is only minimal motion from one projection to the next. One of the most effective methods of respiratory gating is to intubate the animal and control the respiration by mechanical ventilation (Hedlund and Johnson, 2002; Namati et al., 2006). This allows projections to be acquired at exactly the same point in each respiratory cycle. For a less invasive approach, the respirations of a freely breathing animal can be monitored using a pressure transducer. The x-ray projections can then be acquired automatically at the same point in the measured respiratory cycle (Badea et al., 2004). This method does not perfectly eliminate respiratory motion, but it is much less invasive than mechanical ventilation and can still resolve features down to ∼150 microns (Namati et al., 2006). Retrospective gating is also possible, in which many projections are acquired rapidly and sorted post-acquisition according to phase of the respiratory cycle. Subsequently, these sorted projections are used for the reconstruction of tomographic images corresponding to each phase of the respiratory cycle (Ford et al., 2007).

**FIGURE 4:**
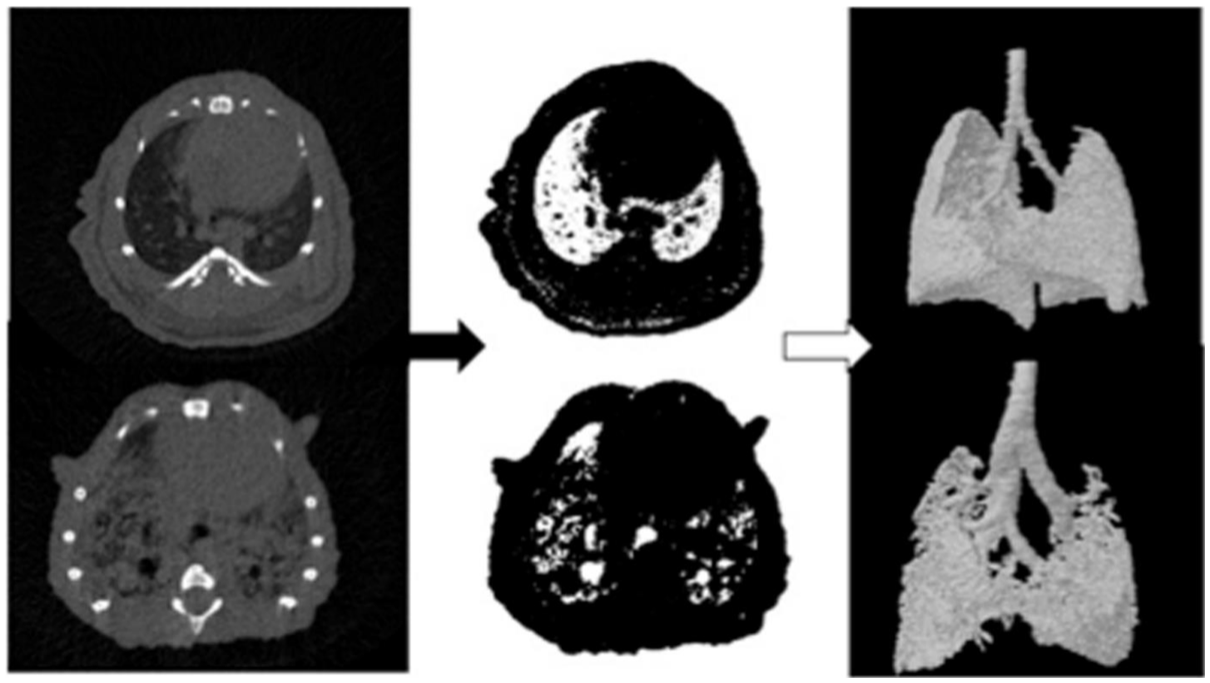
**Automated analysis of lung air volumes for a normal mouse and a mouse with lung fibrosis.** CT cross-sections of the lungs are thresholded to include only those voxels which primarily contain air. These binary images are then converted to 3D volumes to visualize and quantify the aerated lung volumes. The fibrotic lung has significantly reduced air volume compared to the normal lung. Reprinted from (De Langhe et al., 2012) under the Creative Commons Attribution License.

Micro-CT with respiratory gating has been used to study a wide variety of lung diseases. Micro-CT can be used to longitudinally monitor mice for the presence of lung metastases (Li et al., 2006) as well as follow the growth of lung tumors (Hori et al., 2008; Namati et al., 2010; Li et al., 2013a; Rudyanto et al., 2013). The treatment efficacy of chemotherapy (Ueno et al., 2012) or radiation therapy (Perez et al., 2009, 2013; Kirsch et al., 2010) on lung tumors can be measured using micro-CT, and lung injury resulting from radiation therapy can also be assessed (Saito and Murase, 2012). In addition to tumor characterization, micro-CT is also useful for imaging diseases of the lung parenchyma. Mouse models of emphysema created by intra-tracheal instillation of elastase (Postnov et al., 2005; Artaechevarria et al., 2011; De Langhe et al., 2012; Munoz-Barrutia et al., 2012) or exposure to cigar smoke (Sasaki et al., 2015) have been developed and characterized by micro-CT. In emphysema, CT values decrease compared to normal lung due to the loss of soft tissue parenchyma and increased air-trapping. A mouse model of bleomycin-induced lung fibrosis has also been studied extensively by micro-CT (Shofer et al., 2007, 2008; De Langhe et al., 2012) and this model has been used with micro-CT for the preclinical evaluation of drug efficacy (Scotton et al., 2013; Choi et al., 2014; Zhou et al., 2015). In fibrosis, CT values increase due to an expansion of the parenchyma tissue. Lung compliance and lung volume, which are important factors in both emphysema and fibrosis, can also be measured by micro-CT. Animals are mechanically ventilated at multiple pressures and the lung volume at each pressure is measured. The resulting lung pressure-volume curve can be used to calculate lung compliance (Guerrero et al., 2006; Shofer et al., 2007). **Figure 4** shows an example of automatic quantification of lung air volumes using micro-CT in normal mice and in mice with bleomycin-induced fibrosis (De Langhe et al., 2012). Micro-CT has also been used to detect chronic silicosis (Artaechevarria et al., 2010) and acute respiratory distress syndrome (Voelker et al., 2014).

## Micro-Ct Contrast Agents

Because of the lack of inherent contrast for soft tissue imaging, the majority of CT scans make use of high atomic weight contrast agents. In current clinical practice, iodine is the most commonly used element for intravascular CT contrast. Iodine contrast agents are made up of water-soluble aromatic iodinated compounds. These compounds provide effective contrast due to their high atomic number, which produces a strong photoelectric effect. Because CT is relatively insensitive to contrast, high concentrations of contrast agent (up to 400 mg iodine/mL) must be injected in order to produce adequate image enhancement. Clinical CT contrast agents are generally safe, but severe adverse reactions sometimes occur. These adverse reactions are generally divided into two types: allergic reactions and contrast-induced nephropathy (CIN). CIN occurs due to the high osmolality and viscosity of clinical contrast agents and is more common in patients with chronic renal disease (Namasivayam et al., 2006; Tepel et al., 2006; Wang et al., 2007). Iodinated contrast agents are rapidly cleared from the bloodstream by the kidneys (Bourin et al., 1997), so there is only a very short window for imaging after injection. Additionally, these agents quickly distribute from the intravascular to the extravascular space throughout the body. Initially, this provides useful contrast, but after a short time this nonspecific uptake leads to uniform enhancement throughout most of the body. Development and optimization of these small molecule contrast agents continues in order to address some of these limitations, but no breakthroughs have occurred in clinical contrast agents for many years (Lusic and Grinstaff, 2013). This lack of progress is primarily due to the significant hurdle of developing high atomic weight agents that simultaneously demonstrate low toxicity, high efficacy, and low cost.

For small animal imaging, the use of clinical contrast agents is particularly difficult. Small animals have much higher renal clearance rates than humans, so injected contrast agents are rapidly excreted. This can be illustrated for the case of a mouse. In the average adult mouse, blood volume is approximately 1.5–2.0 mL (Diehl et al., 2001), and the glomerular filtration rate (the volume of plasma filtered by the kidneys per time) is approximately 0.4 mL/s (Cervenka et al., 1999). Therefore, the whole mouse blood volume is filtered by the kidneys in less than 5 s. Consistent with this filtration rate, it has been shown that clinical iodine contrast agents drop to undetectable levels in the bloodstream within 4 s of injection in a mouse (Lin et al., 2009). This rapid clearance of contrast agent severely limits the useful application of clinical contrast agents in small animals.

**FIGURE 5:**
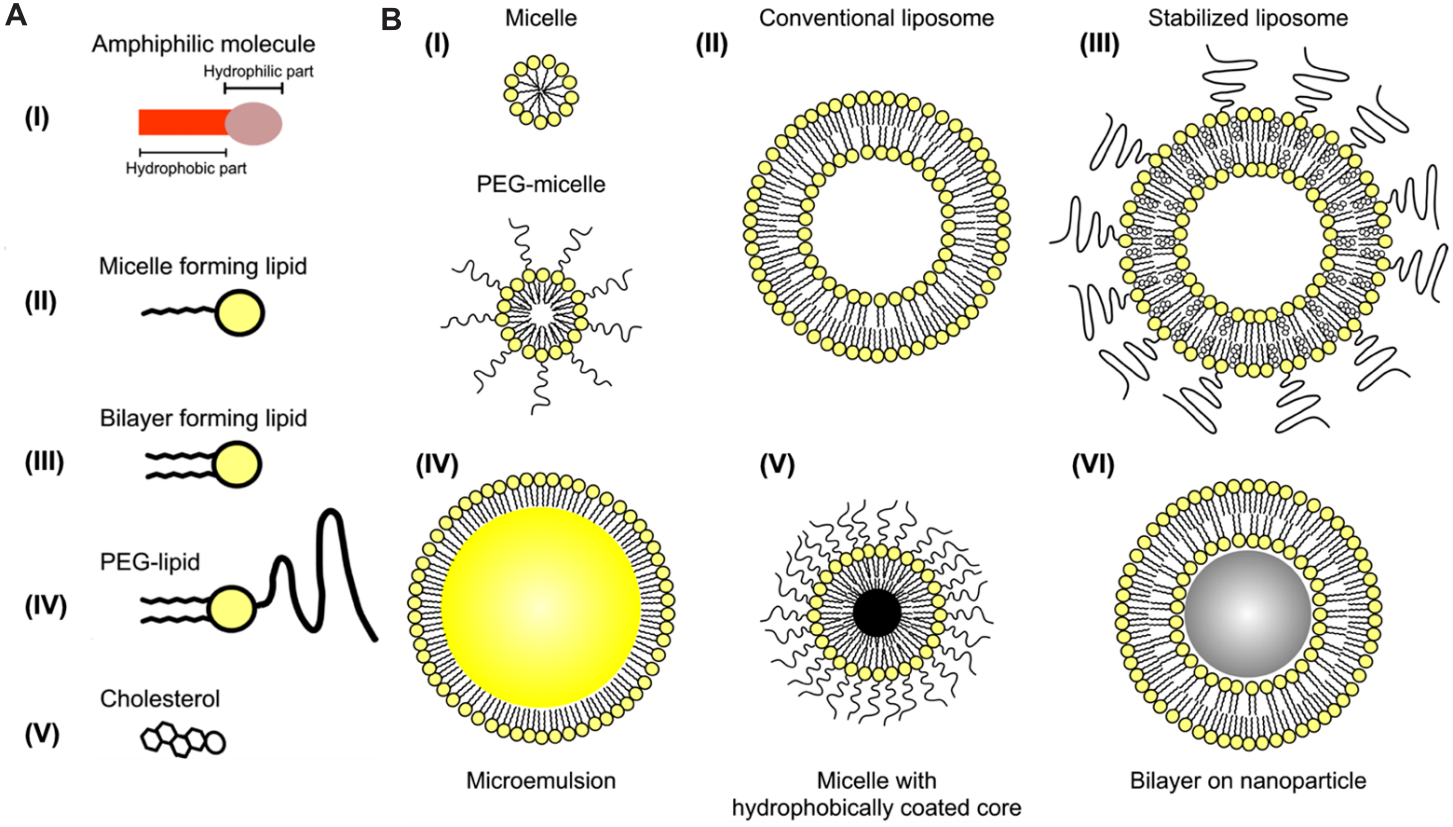
**Iodine-containing nanoparticles.** **(A)** Representation of individual amphiphilic lipids that can be incorporated into nanoparticles. **(B)** Representation of several configurations of self-assembling nanoparticles based on amphiphilic lipids. Iodine can be incorporated into the hydrophobic portion of the micelle, within the non-polar core of an emulsion, or within the aqueous core of liposomes. Reprinted with permission from (Mulder et al., 2006).

To overcome the rapid clearance of traditional contrast agents, blood pool contrast agents have been developed which exhibit prolonged blood residence time and stable enhancement for minutes to hours. Blood pool agents are made up of a wide variety of high molecular weight compounds or nanoparticles that avoid renal clearance due to their large size. Iodine-based blood pool agents include iodine-containing polymers (Galperin et al., 2007; Aviv et al., 2009), micelles (Trubetskoy et al., 1997; Torchilin et al., 1999), emulsions (de Vries et al., 2010; Hallouard et al., 2013; Li et al., 2013b), and liposomes (Krause et al., 1993; Petersein et al., 1999; Mukundan et al., 2006; Ghaghada et al., 2011). A schematic demonstrating the configuration of several iodine-containing nanoparticle agents is shown in **Figure 5.** Historically, these iodine nanoparticles have been the most used contrast agents for micro-CT imaging. The development and use of these iodine-containing blood pool contrast agents have been reviewed elsewhere (Hallouard et al., 2010; Annapragada et al., 2012; Cormode et al., 2014; Li et al., 2014). Some iodine-containing blood pool agents are commercially available for small animal research, including *Fenestra*^®^ (MediLumine, Montreal, QC, Canada) and *Exia^TM^* (Binitio Biomedical, Inc., Ottawa, ON, Canada), and *Exitron^TM^ P* (Miltenyi Biotec, San Diego, CA, USA).

Over the past several years, metal nanoparticle contrast agents have been developed incorporating a wide variety of elements. The most commonly used metal nanoparticles for micro-CT consist of gold. Gold nanoparticles produce greater CT enhancement than iodinated contrast agents because of the high atomic number of gold (*Z* = 79) compared to iodine (*Z* = 53). Gold nanoparticles are particularly promising for *in vivo* imaging applications because gold is extremely inert and gold nanoparticles can be readily modified with surface-linked molecules to render them biocompatible (Li et al., 2012). Bismuth is another promising element for use as contrast agent because it is plentiful, inexpensive, and has a high atomic number (*Z* = 83). Multiple formulations of bismuth nanoparticles have been proposed for use as CT contrast agents (Rabin et al., 2006; Ai et al., 2011; Perera et al., 2011; Swy et al., 2014). Nanoparticles for micro-CT have also been developed using other metals, including bismuth, barium, tantalum, silver, gadolinium, ytterbium, and thorium (Jakhmola et al., 2012). Some metal nanoparticle contrast agents are commercially available, including the gold nanoparticle agent AuroVist^TM^ (Nanoprobes, Inc., Yaphank, NY, USA) and the barium nanoparticle agent *Exitron^TM^ Nano* (Miltenyi Biotec).

Surface conjugation is important for nanoparticle contrast agents, because bare nanoparticles adsorb serum proteins and are readily recognized and cleared by the immune system. A variety of molecules can be added to the nanoparticle surface to decrease nanoparticle clearance, but the most common modification strategy is the addition of polyethylene glycol (PEG; Jokerst et al., 2011). Surface PEGylation significantly increases nanoparticles’ blood residence time, which allows them to be used as blood pool contrast agents. Nanoparticles’ blood residence time and biodistribution are also heavily influenced by their size and shape, with smaller nanoparticles tending to have longer blood residence times.

## Applications Of Contrast-Enhanced Micro-Ct

The development of nanoparticle contrast agents has opened the door for many exciting applications in small animal imaging. While imaging applications using low molecular weight contrast agents have been limited, blood pool contrast agents have now been used for a wide range of imaging applications. Important modern applications for contrast-enhanced micro-CT in small animals include imaging of the vasculature, heart, abdomen and tumors. Current micro-CT contrast agent research is now focused on developing agents with active targeting, multi-modal, or theranostic capabilities.

### Vascular Imaging

**FIGURE 6:**
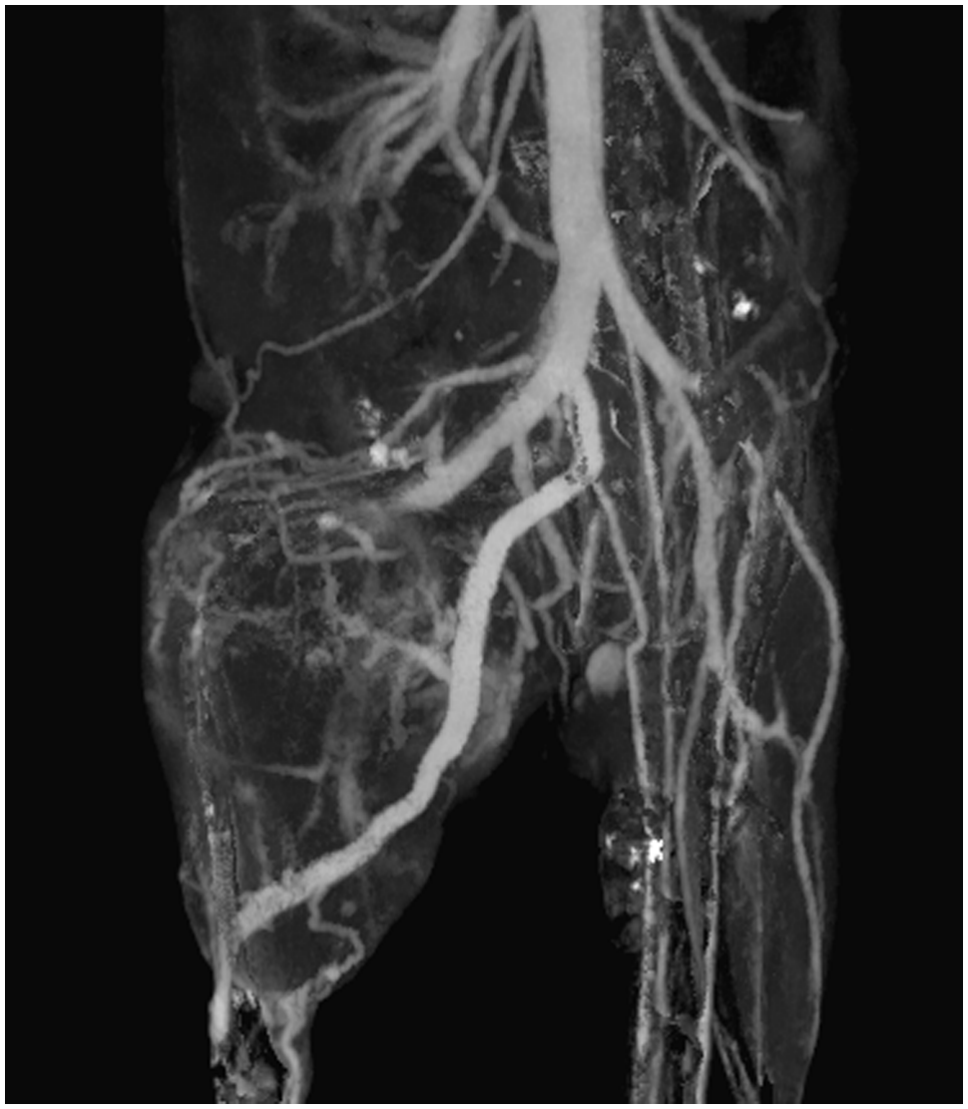
**Coronal maximum intensity projection of intravascular iodine in a mouse with a soft-tissue sarcoma in the right hindlimb.** Micro-CT imaging (88 μm voxel size) was performed immediately after injection of a liposomal iodine contrast agent.

Vascular imaging for micro-CT is done primarily using blood pool contrast agents. Micro-CT scan times must be longer than clinical CT scan times due to the requirement for much higher resolution. Higher resolution implies a need for more x-ray flux, which is achieved with a longer integration time per projection. Early micro-CT scanners required up to an hour to complete a scan. In these cases, low molecular weight contrast agents could not be used for vascular imaging, as they would be cleared from the bloodstream long before the image acquisition was completed. For current micro-CT scanners, scan times of under a minute are now possible. Using these fast protocols, low molecular weight contrast agents have been successfully used for vascular imaging (Kiessling et al., 2004; Badea et al., 2006; Schambach et al., 2010). However, these contrast agents must be either repeatedly or continuously administered over the course of a scan to achieve a constant level of vascular enhancement. This increases the difficulty of imaging and may significantly increase the injected dose of contrast agent. As an alternative to low molecular weight contrast agents, blood pool contrast agents have been successfully used for a variety of vascular applications, including measurements of vascular morphology, diameter, and branching (Vandeghinste et al., 2011), imaging pulmonary vasculature (Johnson, 2007), imaging hepatic vasculature (Chouker et al., 2008), imaging tumor vasculature (Badea et al., 2006; Graham et al., 2008), and measuring vascular permeability (Langheinrich and Ritman, 2006). By providing a constant level of enhancement within the vasculature over a prolonged period of time (minutes to hours), these contrast agents simplify the acquisition of vascular images using micro-CT and allow for a wider range of imaging protocols to be used. **Figure 6** shows an example of vascular imaging. In this study, micro-CT was used with a liposomal iodine contrast agent in order to study the vasculature associated with primary soft tissue sarcomas of the hindlimb (Moding et al., 2013).

### Cardiac Imaging

Cardiac imaging is challenging in small animals due to their rapid heart rate (∼600 bpm for mice). Like respiratory gating (see Lung Imaging), cardiac gating can be used to minimize artifacts due to cardiac motion in the resulting CT images. Cardiac gating can be performed either prospectively (Badea et al., 2005, 2008a, 2011b; Ford et al., 2005; Guo et al., 2011) or retrospectively (Bartling et al., 2007; Song et al., 2007; Badea et al., 2008a, 2011c; Ashton et al., 2014a). In both cases, the ECG of the animal is continuously monitored. In prospective gating, each projection is triggered at a pre-defined point of the cardiac cycle, so that the heart is in the same position in each of the projections. In retrospective gating, projections are acquired rapidly over several rotations and then the timing of the images is compared to the ECG tracing. Each of the images is sorted into projections belonging to different points in the cardiac cycle. Each set of projections can then be compiled together for tomographic reconstruction. Retrospective gating is much more rapid, but produces an irregular angular distribution of projections, which can cause artifacts during the reconstruction process. Because prospectively gated images are acquired over many cardiac cycles, they require several minutes to perform. Many cardiac imaging protocols incorporate both respiratory and cardiac gating to minimize overall thoracic motion during the scan (Badea et al., 2004). We note that intrinsic retrospective gating can also be implemented with cardiac and respiratory motion signals derived from information within each of the acquired projections, thus avoiding the complications of having ECG or respiratory sensors attached to the mouse (Bartling et al., 2008; Johnston et al., 2010; Kuntz et al., 2010).

**FIGURE 7:**
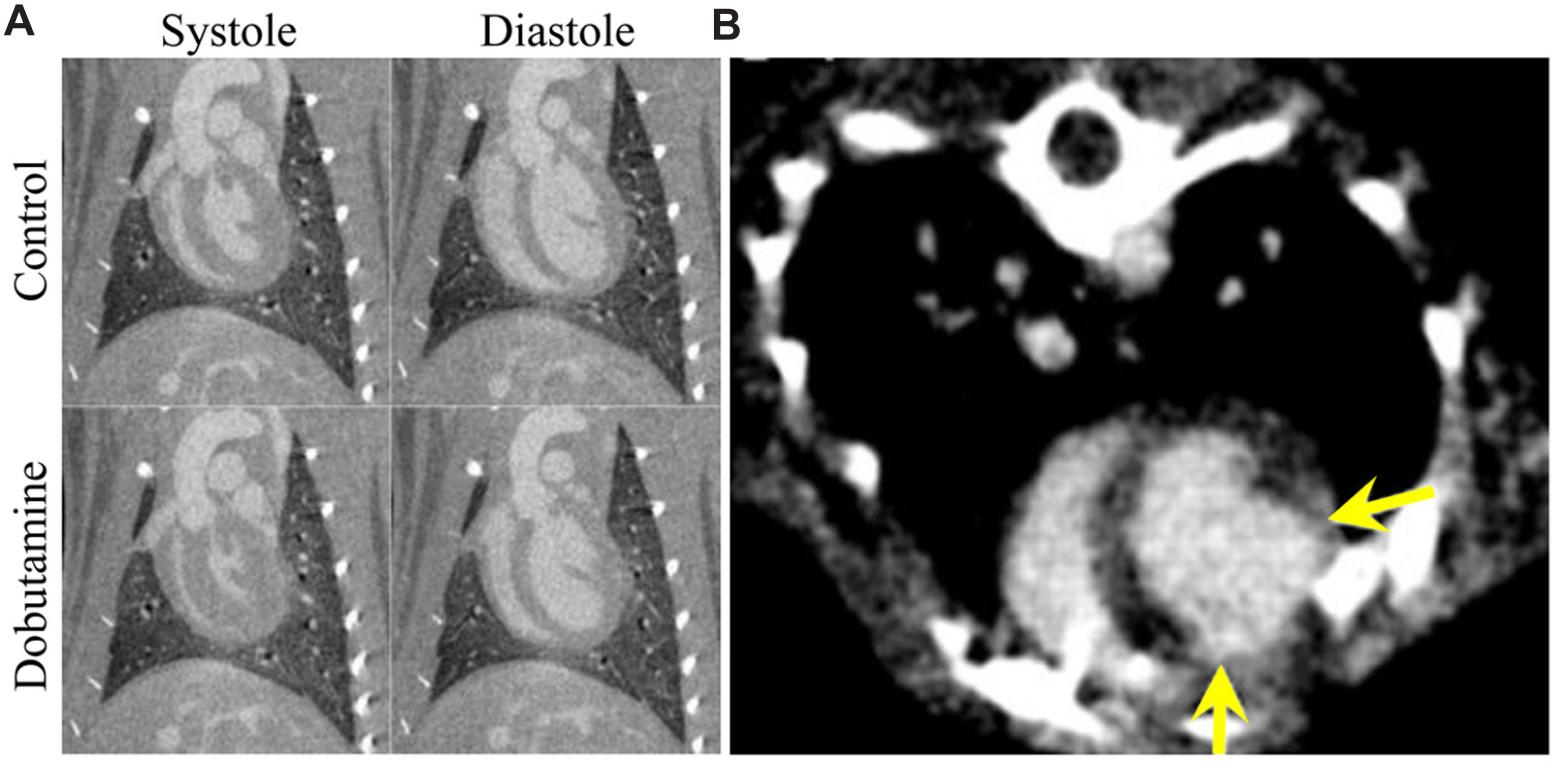
**Cardiac micro-CT imaging.** **(A)** Coronal micro-CT images through the left ventricle of a rat showing the heart in systole (left) and diastole (right) with and without the administration of dobutamine (10 μg/kg/min). End systolic volume is significantly decreased and stroke volume and cardiac output are both significantly increased. End diastolic volume is relatively unchanged after administration of dobutamine. **(B)** An axial image showing myocardial infarction in a rat using delayed hyper enhancement. The yellow arrows show the boundaries of the region of myocardial infarction.

For all cardiac imaging, contrast agents are necessary to differentiate the myocardium from the heart lumen. Because cardiac-gated scans can require several minutes to perform, enhancement of the blood within the heart must remain constant for a prolonged period of time to produce high quality scans. Such imaging is possible with low molecular weight contrast agents by using continuous administration or repeated injections (Sawall et al., 2012), but the vast majority of studies have made use of blood pool contrast agents, which make cardiac-gated CT protocols practical. Because images can be acquired over multiple phases of the cardiac cycle, cardiac micro-CT can produce 4D images of the beating heart. These datasets can be used to measure cardiac function, including ventricular volumes, stroke volume, ejection fraction, wall motion, and cardiac output (Badea et al., 2005, 2007, 2008b, 2011b; Wetzel et al., 2007). Measurements of cardiac function by micro-CT can be used to evaluate the effect of drugs in preclinical studies. Cardiac micro-CT has been used to measure changes in cardiac function as a result of dobutamine-induced cardiac stress (Badea et al., 2011c), as shown in **Figure 7A.** Cardiac micro-CT can also be used to longitudinally measure changes in cardiac function over time. For example, left ventricular remodeling following a coronary ligation-induced myocardial infarction has been tracked by micro-CT (Sheikh et al., 2010). Measurements of cardiac function and infarct size have also been performed in coronary ligation mouse models using either a combination of blood pool agent (Fenestra VC) and a low molecular contrast agent (Nahrendorf et al., 2007) or a blood pool contrast agent (Exia 160) which shows specific uptake in myocardium (Ashton et al., 2014a). An example of micro-CT imaging of myocardial infarction using a delayed hyperenhancement protocol (Nahrendorf et al., 2007) is shown in **Figure 7B.**

### Liver and Spleen Imaging

**FIGURE 8:**
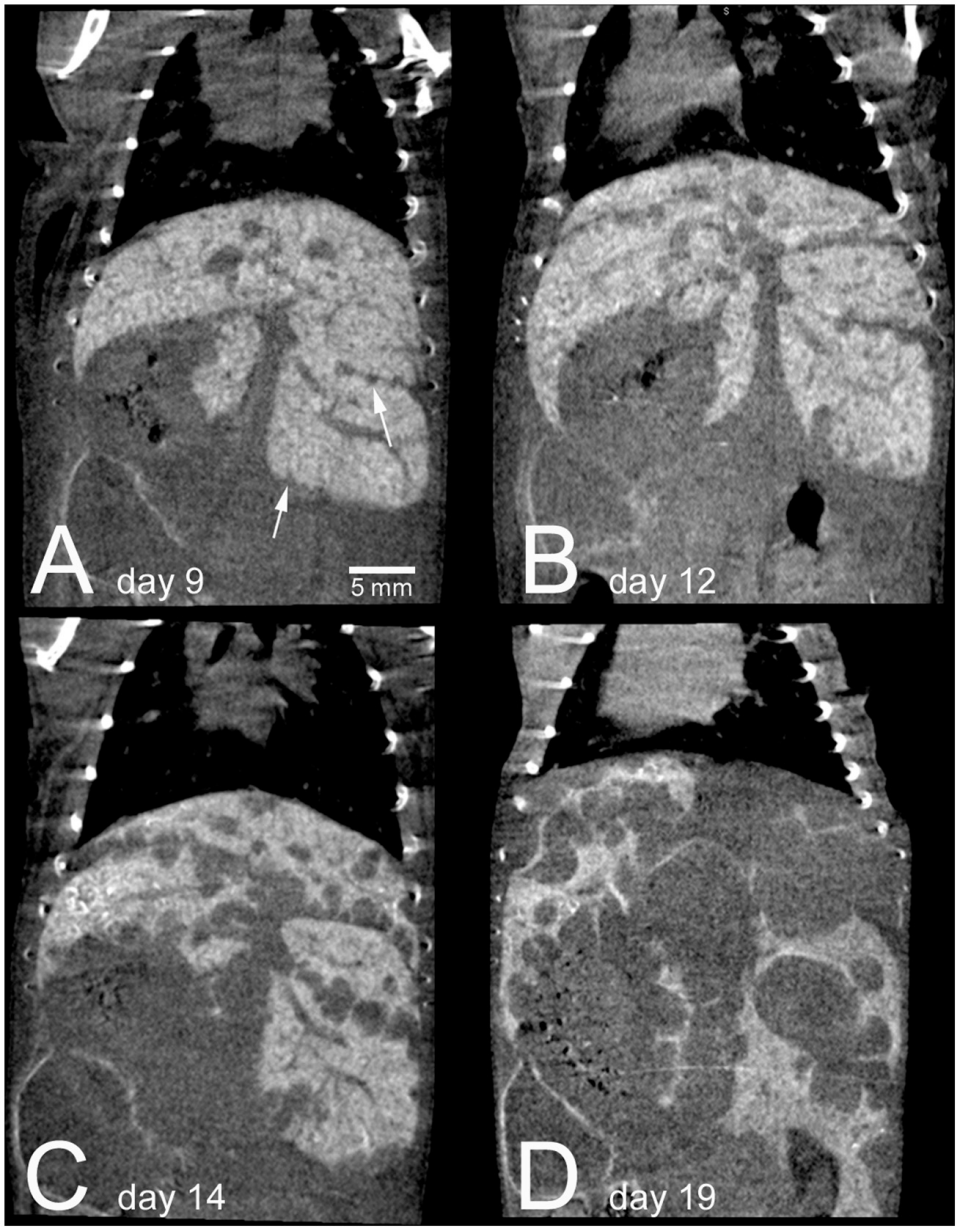
**Longitudinal micro-CT imaging of liver metastases in a mouse following injection of a nanoparticle contrast agent.** A single mouse is shown at 9 days **(A)**, 12 days **(B)**, 14 days **(C)**, and 19 days **(D)** after intrasplenic injection of tumor cells. Normal liver tissue is highly enhancing due to nanoparticle uptake, while the tumor regions show no enhancement. The enhancement remains high within the normal liver over the entire course of the experiment. By day 19, metastatic tumors take up the majority of the liver volume. Reprinted from (Boll et al., 2011) under the Creative Commons Attribution License.

Blood-pool contrast agents, which avoid renal clearance due to their large size (>6 nm), are eventually cleared from the bloodstream by phagocytic cells in the reticuloendothelial system (Moghimi et al., 2001). This clearance occurs primarily in the liver and spleen, which leads to accumulation of contrast in those organs over time. This leads to high enhancement of these organs for liver and spleen-specific imaging. One of the most commonly used micro-CT contrast agents, Fenestra LC, is composed of iodinated phospholipids which are recognized by the ApoE receptor on hepatocytes and internalized in the liver, which provides additional specificity for liver imaging. Because these blood pool contrast agents get taken up by normal-functioning liver and spleen, they can be used to identify necrotic regions (Chouker et al., 2008), liver tumors (Almajdub et al., 2007; Montet et al., 2007; Desnoyers et al., 2008; Graham et al., 2008; Kim et al., 2008; Boll et al., 2011), and spleen tumors (Almajdub et al., 2007), as well as to measure organ volume, quantify hepatic necrosis (Varenika et al., 2013), and determine liver anatomy (Fiebig et al., 2012). **Figure 8** shows longitudinal imaging of liver metastases as they increase in size over time following a single injection of nanoparticle contrast agent (Boll et al., 2011).

### Cancer Imaging

Because tumors generally have the same density as their surrounding tissues, contrast agents are necessary for tumor identification and characterization by micro-CT. The vast majority of cancer imaging studies have been performed using blood pool nanoparticle contrast agents. Nanoparticles tend to accumulate in tumors due to the enhanced permeability and retention (EPR) effect (Maeda et al., 2000; Maeda, 2001). Rapid angiogenesis within a tumor leads to the development of immature, poorly organized, leaky vasculature. Gaps in this leaky vasculature are large enough that nanoparticles (up to 200–300 nm) can readily extravasate into the tumor tissue. Tumors also tend to have very poorly developed lymphatic drainage, so the nanoparticles are not cleared from the tumor once they extravasate. This effect leads to the gradual passive accumulation of nanoparticles in the tumor perivascular space over the course of hours to days. EPR has been widely exploited for both tumor imaging and therapy using nanoparticle agents.

**FIGURE 9:**
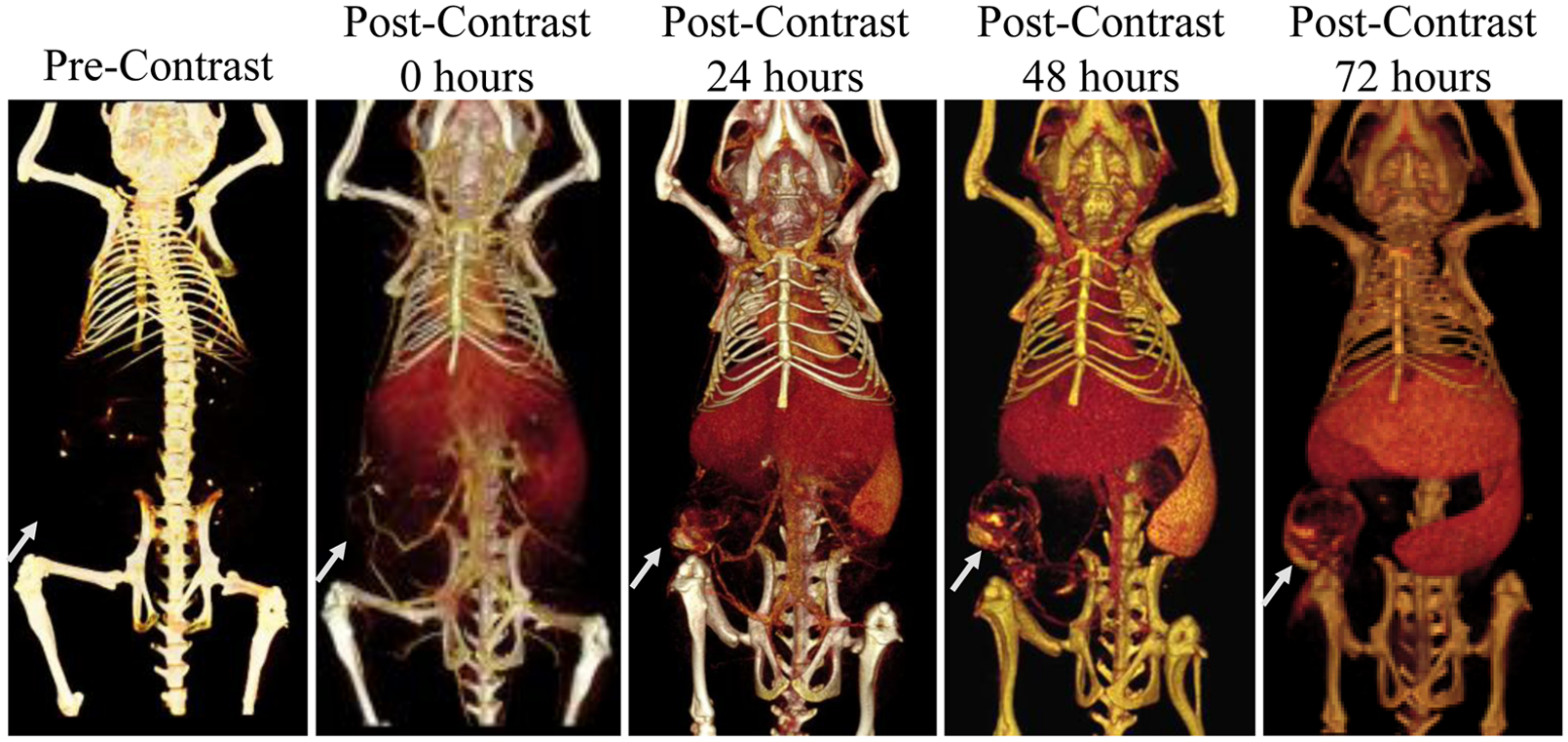
**Longitudinal micro-CT imaging of liposomal iodine biodistribution.** Liposomes slowly accumulate in the subcutaneous tumor, liver, and spleen over the course of 72 h. The white arrow points to the location of the tumor in each image.

Using micro-CT, dynamic biodistribution of contrast agent within small animal tumor models can be tracked. A liposomal iodine contrast agent was used in a rabbit tumor model for contrast agent tracking and biodistribution analysis (Zheng et al., 2009). Quantitative analysis was performed to determine the percent contrast agent uptake within each organ, including the tumor. Liposomal iodine was also used in two mouse models of breast cancer to demonstrate dynamic changes in enhancement within tumor vasculature and tumor parenchyma (Samei et al., 2009; Ghaghada et al., 2011). Immediately after injection, the contrast agent is entirely intravascular, with no significant enhancement within the tumor tissue. This early phase allows for the analysis of tumor vascular morphology, location, and density. After the contrast agent was cleared from the bloodstream, late phase imaging was performed to demonstrate passive accumulation of the contrast agent in the tumors due to EPR. The tumors showed heterogeneous enhancement throughout their volumes, demonstrating spatial heterogeneity in tumor perfusion and vascular permeability. **Figure 9** shows an example of nanoparticle dynamic biodistribution and tumor accumulation for a mouse injected with liposomal iodine (Ghaghada et al., 2011). Immediately after liposome injection, blood vessels are clearly outlined. At later time points, the liposomes accumulate both in the flank tumor and in the liver and spleen. Further studies have been done in mouse xenograft tumor models to carefully map the spatial and temporal distribution of liposome uptake by micro-CT (Ekdawi et al., 2015), which has important implications for nanoparticle-based drug delivery. Measurements of tumor vascular density in early phase imaging and total contrast accumulation in late phase imaging have also been used in two mouse models of lung cancer to differentiate between benign and malignant cancer types (Badea et al., 2012). Iodine-containing nanoparticle contrast agents have also been used for tumor imaging in two other models of lung cancer (Kindlmann et al., 2005; Anayama et al., 2013) and a mouse model of liver cancer (Rothe et al., 2015). Gold nanoparticles have also been used for passive tumor targeting in mouse models of breast and brain cancer (Hainfeld et al., 2006, 2013).

### Active Targeting

**FIGURE 10:**
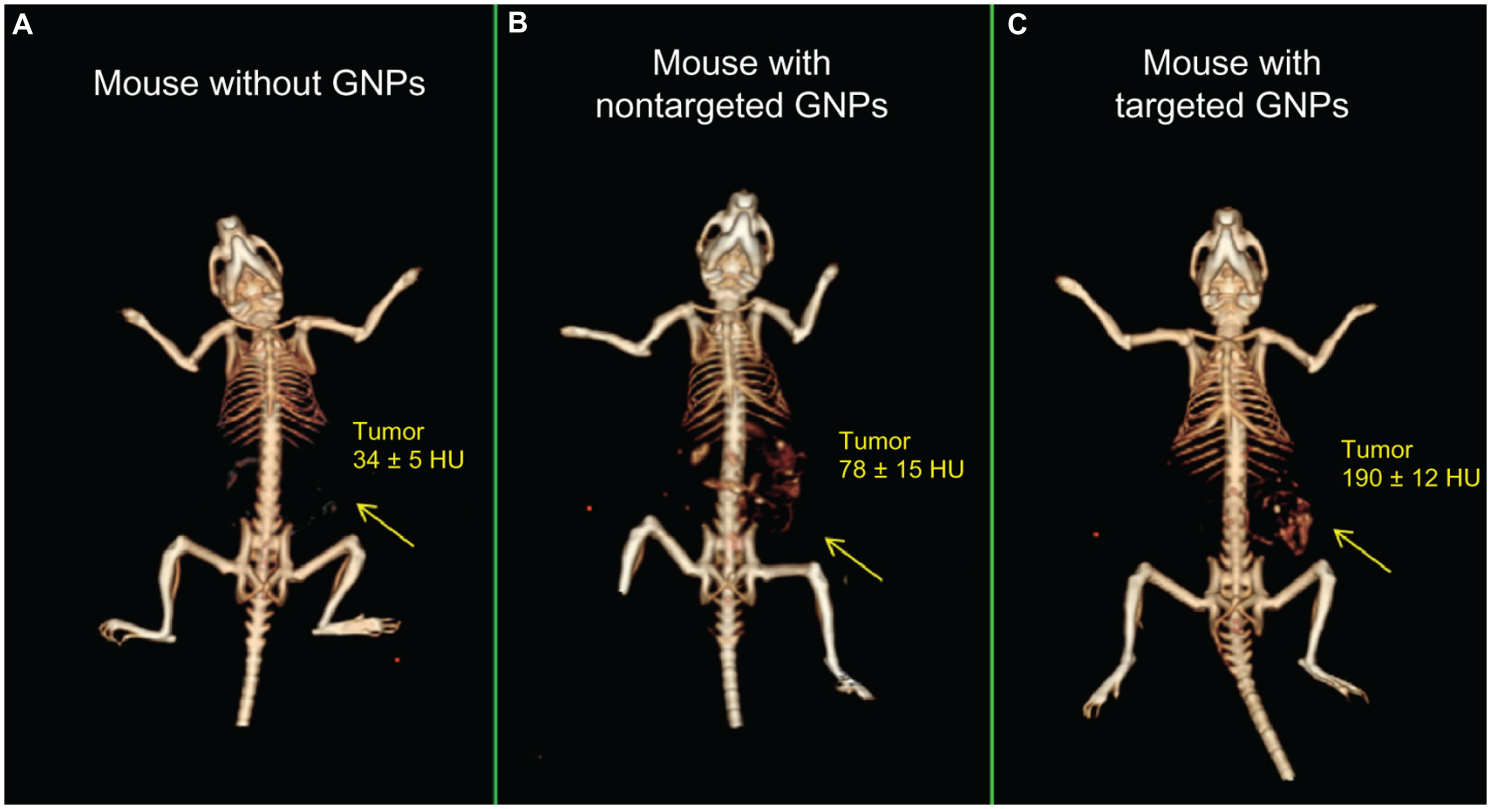
**3D micro-CT reconstructions of mice with EGFR-expressing tumors.** Mice were injected with **(A)** saline, **(B)** non-targeted gold nanoparticles, or **(C)** EGFR-antibody targeted gold nanoparticles. Increased CT enhancement was seen for both types of nanoparticles, but targeted nanoparticles showed significantly higher enhancement than non-targeted controls. Reprinted from with permission from (Reuveni et al., 2011a)

In addition to the passive accumulation of nanoparticles in the reticuloendothelial system or tumors, active targeting of nanoparticles can be accomplished by conjugating specific ligands to the nanoparticle surface which can then link to their binding partners *in vivo* (Erathodiyil and Ying, 2011). Typically, these binding partners are cellular receptors or extracellular matrix proteins that are overexpressed in a pathological condition, so binding is specific to the region of pathology. Potential ligands for conjugation to the nanoparticle surface include antibodies, antibody fragments, other proteins, peptides, aptamers, lipids, carbohydrates, and other small molecules. The use of targeted contrast agents for micro-CT has recently been reviewed (Li et al., 2014). Gold nanoparticles have been used extensively for active targeting due to the ease of gold surface modification via gold-thiol bond formation. Gold nanoparticles have been used as a micro-CT contrast agent for the targeting of multiple tumor markers, including Her2 (Hainfeld et al., 2011), the gastrin-releasing peptide (GRP) receptor (Chanda et al., 2010), the epidermal growth factor receptor (EGFR) (Reuveni et al., 2011b), the folic acid receptor (FAR) (Wang et al., 2013), and tumor microcalcifications (Cole et al., 2014). **Figure 10** demonstrates the use of EGFR-antibody conjugated gold nanoparticles to target an EGFR-expressing subcutaneous tumor. Tumor enhancement was significantly increased with targeted gold nanoparticles compared to non-targeted gold nanoparticles (190 HU vs. 78 HU). Gold nanoparticles have also been used for CT imaging of lymph nodes by targeting CD4 (Eck et al., 2010), imaging of inflammation by targeting intravascular E-selectin (Wyss et al., 2009), imaging of atherosclerosis by targeting fibrin (Winter et al., 2005), imaging of myocardial scars by targeting collagen (Danila et al., 2013), and imaging of other cardiovascular disease (Ghann et al., 2012). In addition to targeting by the surface conjugation of a ligand, some nanoparticles have inherent targeting abilities due to their nanoparticle chemistry. Gold nanoparticle encapsulated within HDL particles are naturally recognized by HDL receptors and taken up in atherosclerotic plaques (Cormode et al., 2010). Exia-160 consists of iodinated molecules which can be fully metabolized by the body, and therefore the contrast agent accumulates in metabolically active tissues, including the myocardium and brown adipose tissue. This effect has been used to discriminate between healthy and infarcted myocardium (Ashton et al., 2014a).

Targeted CT imaging can also be accomplished by labeling cells with nanoparticle contrast agents. Cell labeling with nanoparticles has been successfully used for MRI and other imaging modalities, but has only recently been demonstrated for CT (Betzer et al., 2014). In this study mesenchymal stem cells were labeled with gold nanoparticles prior to injection into a rat model of depression. Cell migration into depression-related bring regions was successfully tracked up to 1 month post-transplantation using micro-CT. The continued development of CT contrast agents for targeted imaging and cell tracking will improve the specificity of CT imaging for a wide range of pathologies and cell therapies and will make molecular imaging with CT a reality.

### Multi-modality Imaging

**FIGURE 11:**
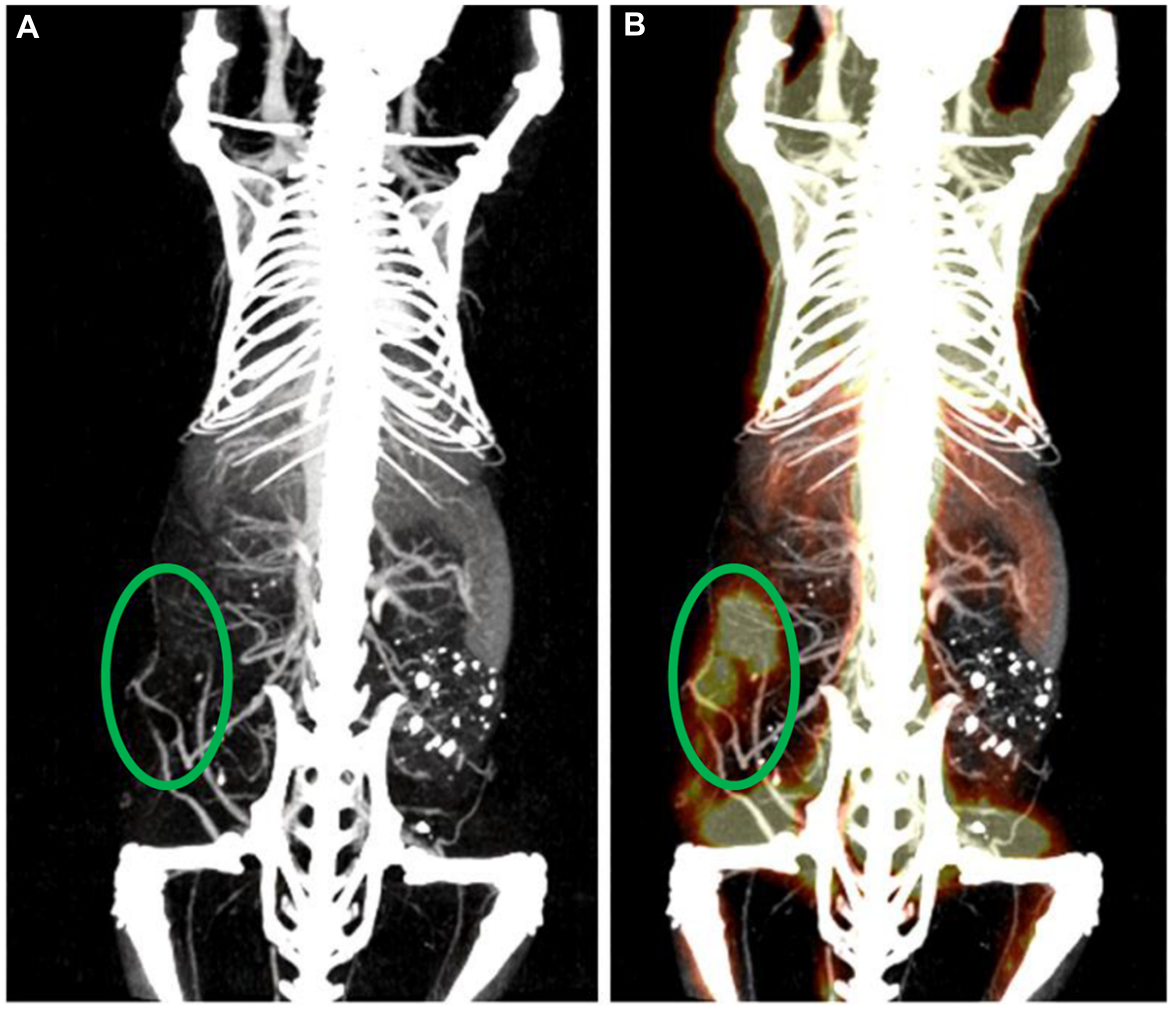
**Multi-modal micro-CT/micro-PET imaging.** **(A)** Maximum intensity projection rendered micro-CT image acquired 1 h post-administration of PEGylated liposomal-iodixanol. **(B)** The overlaid PET/CT image shows the metabolically active tumor (green ellipse).

Micro-CT can also be combined with other imaging modalities in order to better study molecular and anatomical information simultaneously. A micro-CT system can be combined with single photon emission computed tomography (SPECT), positron emission tomography (PET), or fluorescence molecular tomography (FMT) into a single unit (Goertzen et al., 2002; Liang et al., 2007). SPECT, PET, and FMT are all highly sensitive, so targeted molecular imaging with radio-labeled or fluorescently labeled small molecules or biomolecules is readily accomplished. However, these modalities are all limited by poor spatial resolution and poor anatomical imaging. By combining these systems with micro-CT, high resolution anatomical images can be co-registered with molecular images to produce highly useful datasets. Combining micro-SPECT and micro-PET with micro-CT can also improve the image quality of the resultant SPECT and PET images by allowing for attenuation correction (Chow et al., 2005; Hwang and Hasegawa, 2005). **Figure 11** shows a combined micro-CT/micro-PET image for a tumor-bearing mouse soon after injection of both liposomal iodine and ^18^F-fluorodeoxyglucose (FDG) (Badea et al., 2011a). The micro-CT image provides high resolution anatomical detail to give context to the tumor signal seen in the micro-PET image.

A second application of multi-modal imaging which has gained much attention recently is the use of agents that produce contrast for multiple imaging modalities simultaneously. Thus, multiple imaging modalities can be used after injection of a single contrast agent. This helps to improve registration between the different modalities, and increase the amount of information gained from hybrid imaging systems. Many different formulations of multi-modal contrast agents have been developed, and the development of these agents has been reviewed previously (Key and Leary, 2014). Combined CT/MR contrast agents have been developed using gadolinium chelates conjugated to gold nanoparticles (Alric et al., 2008) or gold nanoshells (Coughlin et al., 2014), liposomes containing both gadolinium and iodine-based contrast agents (Zheng et al., 2006), and iron oxide core nanoparticles surrounded by either a gold shell (Carril et al., 2014) or a mesoporous silica shell filled with iodinated oil (Xue et al., 2014). A combined CT/SPECT agent has been developed using a dendrimer linked to both iodinated organic molecules and SPECT agent chelators (Criscione et al., 2011). A combined PET/CT agent has been demonstrated using gold nanoparticles conjugated to both glucose and ^18^F-FDG for targeting of metabolically active tumors (Roa et al., 2012; Feng et al., 2014). All of these formulations have been successfully tested *in vivo* with multi-modal small animal imaging.

### Theranostics

**FIGURE 12:**
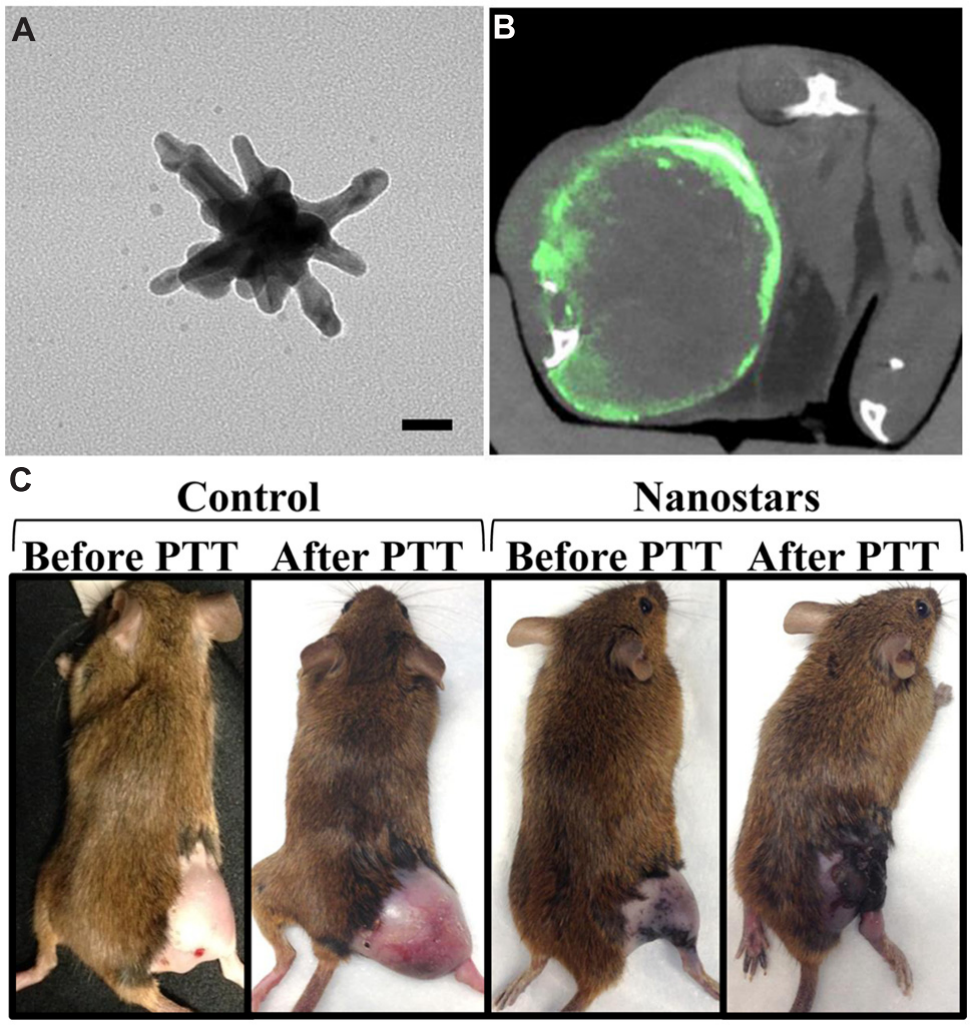
**Theranostic gold nanostars for micro-CT imaging and photothermal therapy.** **(A)** TEM image of gold nanostar (scale bar – 20 nm). **(B)** Micro-CT axial section through the soft tissue sarcoma on a mouse hindlimb following gold nanostar injection. Green represents gold concentration (windowed from 2 to 10 mg/mL). **(C)** Photothermal therapy after injection of either gold nanostars or saline. The mice receiving gold nanostars showed complete remission of their sarcoma, while the control mice had continued rapid tumor growth.

Another exciting topic of current research is the development of theranostic nanoparticles – nanoparticles that can be used for both therapy and diagnostic imaging. Many nanoparticles used as micro-CT contrast agents can easily be adapted to incorporate therapeutics or act directly as a therapeutic agent themselves. Gold nanoparticles, for example, have the inherent ability to increase the effectiveness of radiation therapy, because they absorb therapeutic x-rays efficiently and then release that energy to the surrounding tissues. This can significantly increase the locally delivered dose in regions of high nanoparticle concentration. This has been used by several groups to effectively treat cancer in multiple animal models (Hainfeld et al., 2004, 2008, 2010, 2013, 2014; Jeremic et al., 2013; Park et al., 2015; Wolfe et al., 2015). Gold nanoparticles also exhibit high absorbance of light at their surface plasmon resonance wavelength, which can be tuned by altering the shape and size of the nanoparticle. For many gold nanoparticle shapes (i.e., nanorods, nanoshells, nanostars), this plasmon resonance occurs in the near infrared region, which is optimal for use with photothermal heating. In photothermal heating, nanoparticles convert laser light into heat, which leads to local hyperthermia. This can be used for tumor ablation when nanoparticles are accumulated within a tumor. The use of nanoparticle for combined CT imaging and photothermal therapy has been recently reviewed (Curry et al., 2014). Gold nanorods (Huang et al., 2011) and hollow gold nanoshells (Park et al., 2015) have both been used for combined CT imaging, radiation therapy, and photothermal therapy. **Figure 12** shows a gold nanostar theranostic probe which was used for CT imaging and photothermal therapy in a mouse model of primary soft tissue sarcoma (Liu et al., 2015). This probe showed high tumor accumulation and CT enhancement as well as effective tumor ablation following photothermal therapy. Therapeutics can also be incorporated into nanoparticles by direct conjugation to the nanoparticle surface or by co-encapsulation of the therapeutic with the imaging agent (e.g., within the aqueous core of a liposome). Both methods have been used for the addition of therapeutic radioisotopes or chemotherapy drugs to nanoparticle contrast agents (Chen et al., 2014; Lu, 2014; Ryu et al., 2014; Zhu et al., 2014).

## Future Directions – Spectral Ct

Much effort has been made to overcome the low contrast sensitivity inherent in CT imaging. The primary method, as discussed above, is to add large amounts of an exogenous contrast agent. However, significant developments have also been made in imaging system design which can potentially improve CT image contrast. One of the most promising recent developments in CT has been the use of spectral information to improve contrast discrimination. In traditional CT imaging, the overall attenuation of x-ray intensity is measured by the detector, but the detected x-rays are not spectrally resolved. The spectrum of transmitted x-rays is important because the absorption of x-rays by different materials is highly dependent on x-ray energy, so the transmitted x-ray spectrum depends on what materials are present along the x-ray path. Therefore, there is a significant amount of information that can be gained by including spectral data in the CT reconstruction process. Based on differences in x-ray absorption, multiple materials can be differentiated and quantified within a single scan using spectral CT.

There are two primary methods used to obtain spectral CT data. The first method, dual-energy (DE) CT, uses x-ray sources with two different energy spectra and traditional energy integrating x-ray detectors. The second method uses a single x-ray source but has energy-resolving detectors (photon counting detectors) that can measure the energy of each detected photons. DE CT is currently used clinically and has been successful in improving imaging for a variety of applications (Jepperson et al., 2013; Aran et al., 2014; Marin et al., 2014; Mileto et al., 2014; Ohana et al., 2014; Paul et al., 2014; Bongartz et al., 2015)

### Dual-energy CT

**FIGURE 13:**
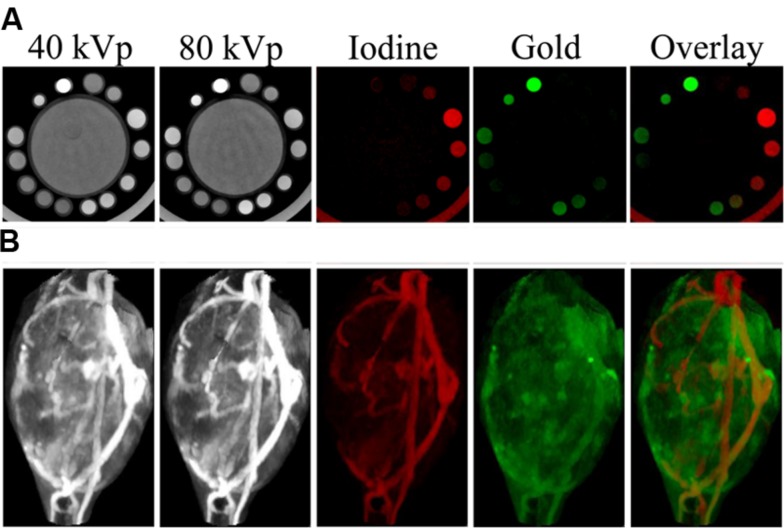
**Dual energy micro-CT material decomposition.** **(A)**, *In vitro* phantom consisting of a large tube of water surrounded by vials containing gold, iodine, or a mixture of the two. **(B)**, *In vivo* imaging of gold nanoparticles and iodine-containing liposomes within a mouse soft tissue sarcoma. The iodine (shown in red) and gold (shown in green) maps are the result of dual energy decomposition. In both cases, the decomposition was able to successfully differentiate the signals from the gold and iodine contrast agents.

DE CT can use either a single x-ray source which rapidly switches between two tube voltages or two separate sources (offset from one another by 90°) that each operate at a unique voltage. In either case, x-ray projections are acquired at each rotation angle using both x-ray sources. Additionally, a double-layer or “sandwich” detector is sometimes used to separate low and high energy x-rays. In DE CT, a complete CT dataset is acquired for two different x-ray energy spectra. Most of a patient’s body appears the same on both images, because absorption of x-rays by low atomic weight materials, which is primarily due to Compton scattering, is very weakly dependent on x-ray energy. However, the photoelectric effect in high atomic weight materials is highly dependent on x-ray energy. Therefore, the attenuation coefficient of high atomic weight materials (calcium in bone, iodine, gold) will depend on the energy spectrum of the incident x-rays. This effect is particularly pronounced if the two energy spectra fall on either side of the K-edge for one of the materials. Because there is a large increase in attenuation at energies above the K-edge (see **Figure 2B**), this leads to a large difference in signal between the two scans. By combining data from the two energy sets, these high Z materials can be differentiated from one another and quantified. This process is demonstrated in **Figure 13A**, which shows scans of an *in vitro* phantom containing vials of water, gold, iodine, or a mixture of gold and iodine. Scans at two different energies were simultaneously acquired. These two scans were then mathematically decomposed into a map of iodine concentration and a map of gold concentration (Clark et al., 2013). We note that although the K-edges have helped with the separation between iodine and gold, we are not able to deliver true K-edge imaging as is possible with synchrotron mono-energetic beams.

Spectral separation using DE CT is somewhat limited by our ability to minimize the overlap of x-ray spectra using polychromatic sources. Although the peak tube voltage can be changed over a wide range, the average energy of the resulting spectrum does not change significantly, as was shown for the two energy spectra in **Figure 2A.** The separation between the two energy spectra can be improved by applying additional filtration to the x-ray tubes, which can preferentially remove low energy photons and further increase the average energy of the x-ray spectrum. The other limitation for DE CT is its ability to discriminate between closely related elements. Discrimination of two elements using DE CT is best when there is a large difference in their attenuations at the two x-ray energies. This works very well for elements with widely different k-edges (gold and iodine), but does not work for elements with very similar k-edges (barium and iodine). By careful selection and design of contrast agents, this limitation can be avoided.

Although DE CT is commonly used in the clinic, its use has been limited to date in preclinical micro-CT imaging. The primary challenge with translating CT to micro-CT is the significant increase in resolution. Because voxel size is much smaller, the noise is much higher for micro-CT than for clinical CT. This could be improved by significantly increasing the number of x-ray photons delivered in order to get the same photon flux through each voxel. However, the radiation dose must be limited for *in vivo* studies, so noise cannot be decreased to the levels seen in clinical scans. This presents a problem for DE reconstruction, because the mathematical decomposition of multiple materials depends on having high quality (low noise) measurements of attenuation at each voxel. High levels of noise make material decomposition inaccurate. By minimizing scatter during acquisition and applying post-acquisition image processing strategies, beam hardening and noise can be reduced to allow for successful DE decomposition. It has been shown that applying joint domain bilateral filtration (an edge-preserving, smoothing filter that incorporates data from both energy sets) prior to DE decomposition significantly improves the DE decomposition accuracy, precision, and limits of detectability (Clark et al., 2013). The mean limits of detectability for each element were determined to be 2.3 mg/mL (18 mM) for iodine and 1.0 mg/mL (5.1 mM) for gold, well within the observed *in vivo* concentrations of each element (I: 0–24 mg/mL, Au: 0–9 mg/mL) and a factor of 10 improvement over the limits without post-reconstruction joint bilateral filtration. *In vitro* testing of this method using imaging phantoms containing both gold and iodine is shown in **Figure 13A** (Clark et al., 2013). Using this method, DE micro-CT has been used successfully for a variety of applications in mice. DE CT was used for atherosclerosis imaging to differentiate liposomal iodine accumulated in plaque macrophages from calcium within the plaque (Bhavane et al., 2013). Iodine accumulated within the myocardium has been separated from other soft tissues and from calcium in the bone for imaging of myocardial infarction (Ashton et al., 2014a). DE CT has been used to separate gold nanoparticles accumulated within soft-tissue sarcomas (Clark et al., 2013) or primary lung tumors (Ashton et al., 2014b) from liposomal iodine within the vasculature. Images of the decomposed gold and iodine maps for a soft-tissue sarcoma are shown in **Figure 13B.** In these studies, the simultaneous measurement of two different nanoparticle concentrations was used to calculate tumor vascular density and vascular permeability. Validation of the calculated results was performed using histology and *ex vivo* measurements of tissue gold and iodine concentrations (Ashton et al., 2014b). In two additional studies, DE CT was used to assess vascular changes following radiation therapy. In the first, the increase in vascular permeability in a soft-tissue sarcoma was determined by measuring accumulation of liposomal iodine (Moding et al., 2013). In the second study, cardiac injury following radiation therapy was assessed using gold nanoparticles and liposomal iodine (Lee et al., 2014). Cardiac-gated CT imaging was performed to obtain a DE decomposition of the myocardium at each phase of the cardiac cycle. This data was used to assess both extent of cardiac injury and change in cardiac function.

**FIGURE 14:**
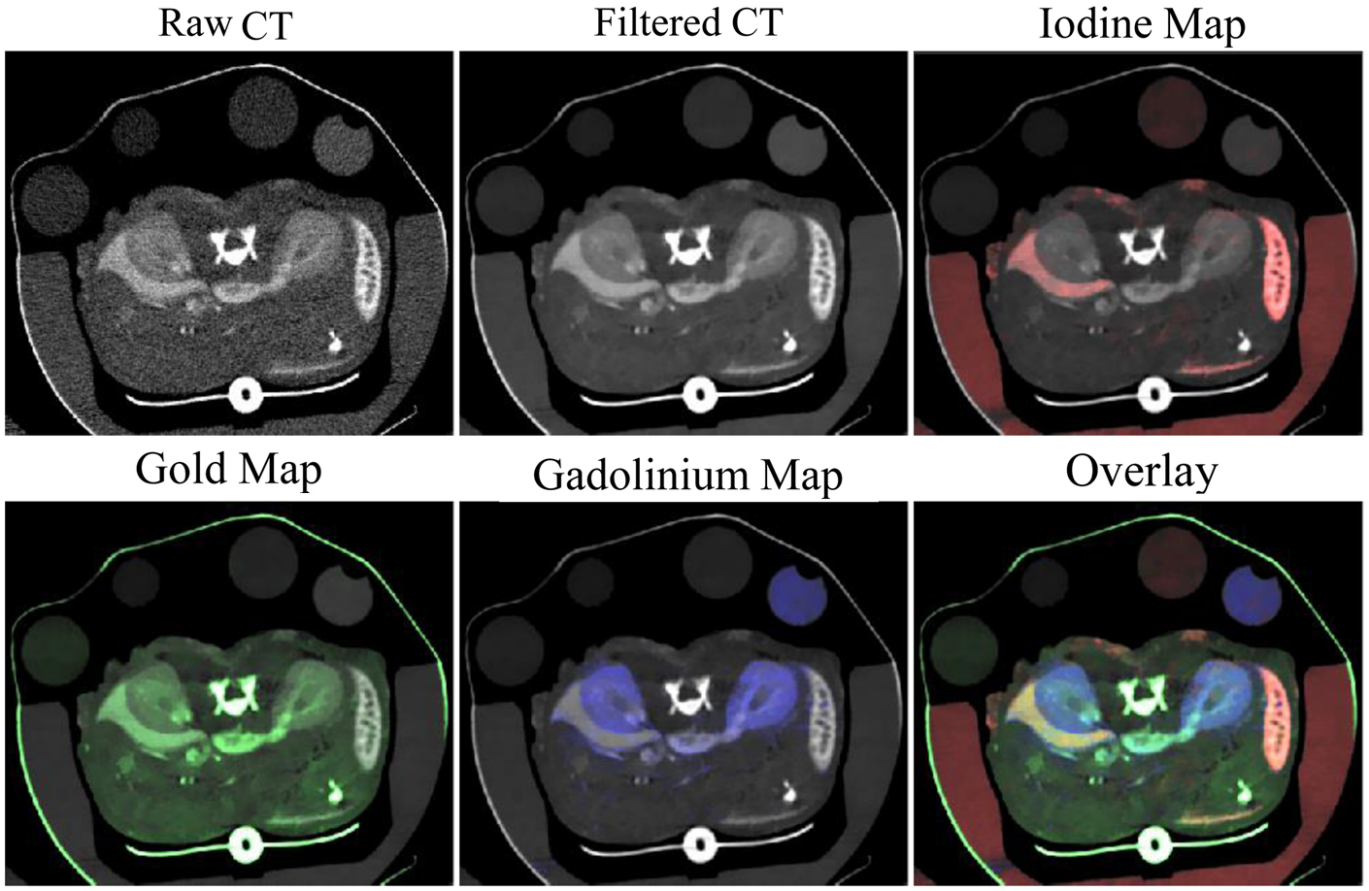
**Three-energy micro-CT imaging in a mouse.** Liposomal iodine was injected 72 h before imaging. Gold nanoparticles and low molecular weight gadolinium were injected immediately before imaging. Images were acquired at three energies, filtered, then separated into maps of iodine (red), gold (green), and gadolinium (blue) concentration.

Our group has recently also demonstrated triple-energy micro-CT for the differentiation of three materials: gold, iodine, and gadolinium. Using a novel algorithm called spectral diffusion (Clark and Badea, 2014), these three materials were successfully separated and quantified both in an *in vitro* phantom and *in vivo.* **Figure 14** shows *in vivo* images with decomposed concentration maps depicting liposomal iodine accumulated within the liver and spleen, gold nanoparticles within the vasculature, and a low molecular weight gadolinium contrast agent in the kidneys. Dual and triple-energy CT have the potential to be particularly useful with targeted contrast agents, so that contrast agents with multiple different targets can be co-injected and individually quantified using a single scan.

### Photon Counting X-ray Detectors

The alternative to DE CT is the use of energy-resolving photon-counting x-ray detectors (PCXDs) for spectral CT imaging. The PCXDs acquire data for each projection using multiple energy bins. These detectors directly convert photons to a digital signal, which decreases the noise that is inherent in traditional energy-integrating detectors (Schirra et al., 2014). Each photon that is counted by the detector is assigned into one of the energy bins, which provides an approximation of the energy spectrum of the transmitted x-rays. Energy bins can be chosen to include regions of the spectrum above and below the K-edge of the elements of interest. The measured attenuations from each energy bin can then be used to simultaneously solve for the concentration of one or more high atomic weight materials within a single voxel. This method can also be used quantify the contribution of either Compton scattering or the photoelectric effect within any given voxel, which allows accurate separation between signal from soft tissues and signal from high Z materials.

Photon-counting x-ray detectors are not yet used in standard clinical CT imaging, but prototype photon counting CT scanners have been deployed in some research hospitals. It is expected that PCXDs will likely be generally adopted in the clinical realm once the technology has further advanced (Taguchi and Iwanczyk, 2013). The primary drawback of current PCXDs is the relatively low photon count rate for each individual detector. Because it takes a finite amount of time to count a single photon, the hardware can fall behind when photon flux is high. This leads to pulse pileup, which can cause saturation of the detectors and loss of spectral sensitivity and accuracy (Schirra et al., 2014). Clinical CT operates at very high photon flux, so this problem must be resolved before PCXDs can be effectively used clinically. The most obvious solution is to decrease the detector size, which will decrease the flux incident on each detector. However, as detectors become smaller, the charge sharing between detectors increases, which can lead to multiple counts for single x-rays and counts at the wrong energies. This leads to spectral distortions and high noise. Therefore, many researchers are focused on improving both the hardware and reconstruction algorithms necessary for optimal spectral imaging with PCXDs.

**FIGURE 15:**
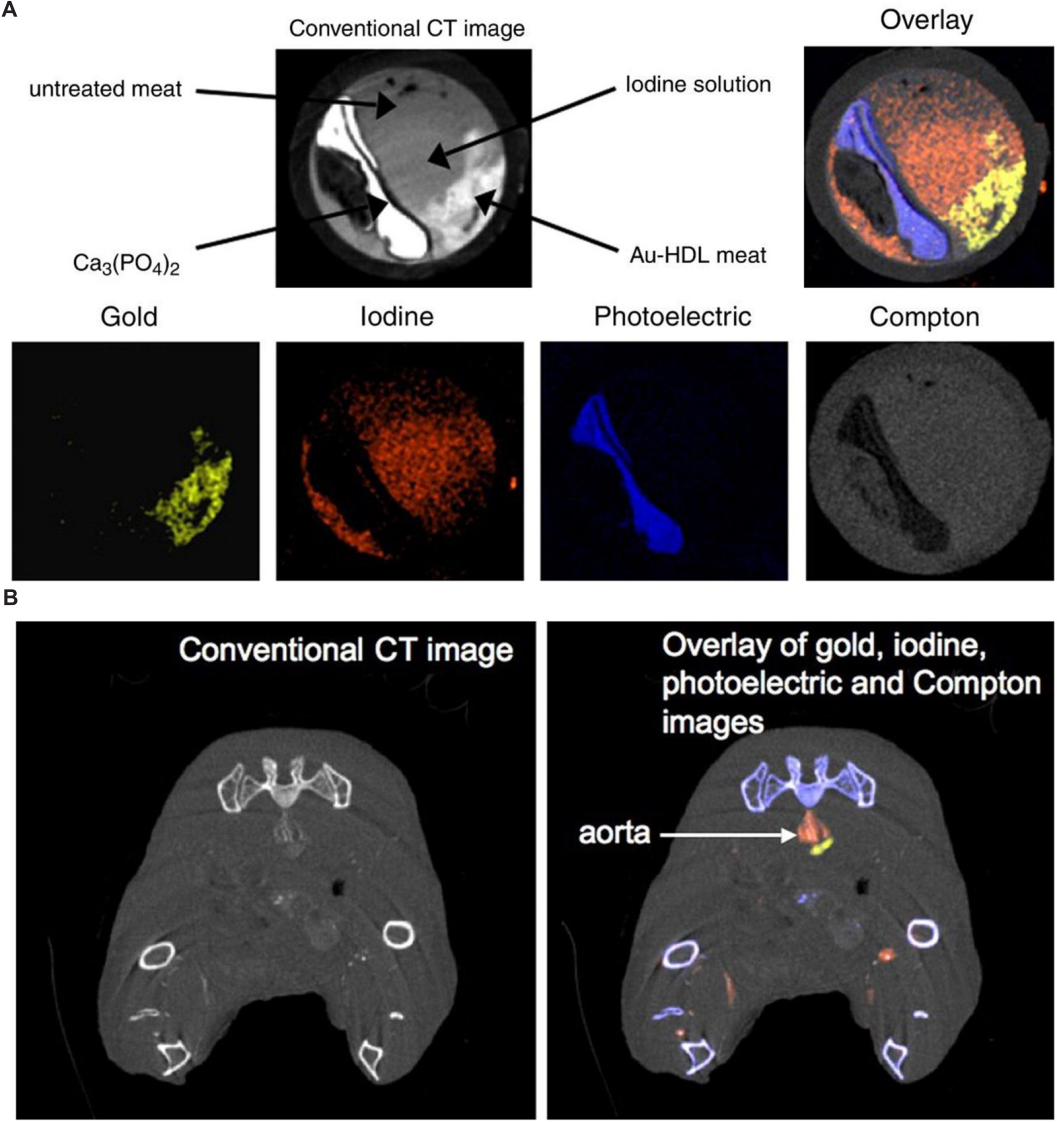
**Spectral micro-CT imaging using photon counting x-ray detectors and HDL-encapsulated gold nanoparticles.** **(A)** In vitro aorta phantom study demonstrating the conventional CT image along with the decomposition of the CT image into gold, iodine, photoelectric, and Compton components. **(B)** *In vivo* imaging of targeted gold nanoparticles and blood pool iodine in a mouse model of atherosclerosis. The iodine (red) can be clearly visualized within the aorta, while the gold signal (yellow) is immediately adjacent to the aorta lumen in the atherosclerotic plaque. Reprinted with permission from (Cormode et al., 2010).

Although PCXDs are still experimental for clinical CT, their use in preclinical small animal studies has been successfully demonstrated. Spectral CT has been used with targeted nanoparticles to image atherosclerotic plaques (Cormode et al., 2010). Gold nanoparticles were encapsulated within high-density lipoprotein (HDL) particles to target plaque macrophages. A preclinical spectral CT system (Phillips Research, Hamburg) was used to differentiate the gold from iodine, calcium, and soft tissues. This analysis was first performed in an *in vitro* aorta phantom, as shown in **Figure 15A.** Spectral CT was used to resolve the signals from gold, iodine and calcium within the tissue phantom matrix. The spectral CT system successfully differentiated the phantom regions containing gold, iodine, and calcium, with very little overlap between the signals. They also tested the targeting of their gold-HDL particles in a mouse model of atherosclerosis. Spectral CT (and subsequent histology) demonstrated that the gold successfully accumulated within the plaques and that gold could be discriminated from iodine, calcium, and soft-tissue *in vivo*, as seen in **Figure 15B.** The same HDL-encapsulated gold nanoparticles have been used along with a blood pool iodine contrast agent to simultaneously image the signals from gold accumulated within lymph nodes, iodine within the blood, bone, and soft tissue (Roessl et al., 2011). Low density-lipoproteins (LDLs) labeled with gold nanoparticles have also been used to image tumors using spectral CT (Allijn et al., 2013). Gold nanoparticles accumulating in lymph nodes after subcutaneous injection have been differentiated from soft tissue and bone (Schirra et al., 2012). Iodine within the vasculature and barium within the gastrointestinal tract have been imaged and differentiated from bone and soft tissue (Anderson et al., 2010). Spectral imaging has also been used to detect novel ytterbium nanoparticles within the vasculature (Pan et al., 2012) and organic bismuth nanocolloids targeted to fibrin-rich clots (Pan et al., 2010). In both cases, spectral CT was used to differentiate contrast agent signal from soft tissue and bone. The primary limitation in all of these studies was that the low photon-count rate limitations of the PCXD system resulted in a long scan time. Because the scan time was so long, the imaging was done after sacrificing the animals in order to prevent motion over the course of the long acquisition. Despite the limitations, these studies demonstrate that spectral CT using a PCXD system has the potential for high quality *in vivo* imaging and material discrimination. Some technical problems remain to be solved, but PCXD systems have great promise for use in both preclinical and clinical CT imaging.

## Radiation Dose Considerations

One of the primary drawbacks of x-ray CT imaging is exposure to radiation. X-ray radiation exposure can lead to biological damage and long-term health effects (Boone et al., 2004). Radiation exposure is particularly important to consider for micro-CT applications, because higher radiation doses are required for high resolution CT scans. Signal-to-noise ratio in CT is inversely proportional to the square root of the number of x-rays passing through each voxel. As voxel size decreases, the number of x-rays necessary to maintain a constant signal-to-noise ratio increases significantly. In planning micro-CT studies, a balance must be made between desired image quality and radiation exposure.

The LD_50/30_ radiation dose in mice (the dose required to kill 50% of mice within 30 days) depends on many factors, but tends to be between 5 and 8 Gy (Ritman, 2004; Carlson et al., 2007). The typical radiation dose for a single micro-CT scan can vary widely and reported values in the literature range from 0.017 Gy to 0.78 Gy (Carlson et al., 2007). Rodents have the ability to repair damage from low doses of radiation (∼0.3 Gy) over the course of several hours (Parkins et al., 1985), so most low dose micro-CT scans should have limited biological impact, even when the same animals are longitudinally scanned over the course of a study. But for higher dose scans, longitudinal imaging can potentially lead to a cumulative dose that could affect biological function (particularly immune function and tumor response) and long-term health (Boone et al., 2004). Therefore, careful consideration must be made to determine the optimal imaging protocol for each individual application to minimize the effects of radiation dose on the experiment. With additional advances in micro-CT technology and reconstruction algorithms, radiation doses should further decrease, which will help to overcome radiation as a limitation of micro-CT imaging.

## Nanoparticle Contrast Agent Safety

Understanding the potential toxicity of nanoparticles is essential in order to apply nanoparticle contrast agents *in vivo* and eventually translate these contrast agents to the clinic. Because blood pool contrast agents are not rapidly cleared from the body by the kidneys, they have much more opportunity to interact with the body and accumulate in various organs. Since each nanoparticle formulation is unique, rigorous toxicity testing must be performed for any proposed contrast agent in order to fully understand its usefulness for both preclinical research and potential clinical translation. For example, there is strong evidence regarding bio-compatibility of gold (Cervenka et al., 1999; Hainfeld et al., 2006; Lin et al., 2009). Gold-based nano-products are now undergoing clinical trials, e.g., colloidal Au-based tumor necrosis factor (CYT-6091, CytImmune, Inc., Rockville, MD, USA) and gold nanoshells (Nanospectra, Inc., Houston, TX, USA). However, there is still uncertainty regarding the toxicity of many of the recently proposed nanoparticle contrast agents; although most of the studies reviewed here have stated that no toxicity has been observed, comprehensive prospective toxicity studies are still required to be performed. Because many nanoparticles can accumulate in the body for up to several months, in depth studies of long term toxicity are particularly important. A better understanding of nanoparticle toxicity is necessary for the further advancement of the field of nanoparticle CT contrast agents.

## Conclusion

Micro-CT has become an extremely important tool in small animal research. Micro-CT produces non-invasive, three-dimensional, high resolution anatomical images, which can provide a wealth of information about normal animal function and pathology. Although x-ray CT is limited by low tissue contrast, developments in contrast agent design show great promise for use in imaging a wide range of organ systems and pathologies. Additional new developments in spectral imaging will further improve the usefulness of micro-CT in acquiring functional and molecular information. This will greatly expand the potential applications for micro-CT in small animal research. The increasing availability and low cost of micro-CT scanners promises to greatly increase the use and impact of micro-CT imaging on small animal studies. Given the common use of mouse models of disease to validate potential drug targets, to assess therapeutic efficacy, and to identify and validate biomarkers of drug efficacy and/or safety, micro-CT with nanoparticle based contrast agents can have far-reaching applications in drug discovery and pharmacology. Continuous development of novel CT/micro-CT imaging technology and contrast agents will serve well drug discovery and result in better medicines. Contrast agents and technology developed for preclinical micro-CT also have the potential to translate to significant improvements in clinical CT imaging.

## Conflict of Interest Statement

The authors declare that the research was conducted in the absence of any commercial or financial relationships that could be construed as a potential conflict of interest.

## References

[B1] Ai K. Liu Y. Liu J. Yuan Q. He Y. Lu L. (2011). Large-scale synthesis of Bi(2)S(3) nanodots as a contrast agent for in vivo X-ray computed tomography imaging. *Adv. Mater.* 23 4886–4891. 10.1002/adma.201103289 21956662

[B2] Allijn I. E. Leong W. Tang J. Gianella A. Mieszawska A. J. Fay F. (2013). Gold nanocrystal labeling allows low-density lipoprotein imaging from the subcellular to macroscopic level. *ACS Nano* 7 9761–9770. 10.1021/nn403258w 24127782 PMC3863599

[B3] Almajdub M. Nejjari M. Poncet G. Magnier L. Chereul E. Roche C. (2007). In-vivo high-resolution X-ray microtomography for liver and spleen tumor assessment in mice. *Contrast Media Mol. Imaging* 2 88–93. 10.1002/cmmi.130 17444558

[B4] Alric C. Taleb J. Le Duc G. Mandon C. Billotey C. Le Meur-Herland A. (2008). Gadolinium chelate coated gold nanoparticles as contrast agents for both X-ray computed tomography and magnetic resonance imaging. *J. Am. Chem. Soc.* 130 5908–5915. 10.1021/ja078176p 18407638

[B5] Anayama T. Nakajima T. Dunne M. Zheng J. Allen C. Driscoll B. (2013). A novel minimally invasive technique to create a rabbit VX2 lung tumor model for nano-sized image contrast and interventional studies. *PLoS ONE* 8:e67355 10.1371/journal.pone.0067355 PMC3696117 23840673

[B6] Anderson N. G. Butler A. P. Scott N. J. A. Cook N. J. Butzer J. S. Schleich N. (2010). Spectroscopic (multi-energy) CT distinguishes iodine and barium contrast material in MICE. *Eur. Radiol.* 20 2126–2134. 10.1007/s00330-010-1768-9 20309554

[B7] Annapragada A. V. Hoffman E. Divekar A. Karathanasis E. Ghaghada K. B. (2012). High-resolution CT vascular imaging using blood pool contrast agents. *Methodist Debakey Cardiovasc. J.* 8 18–22. 10.14797/mdcj-8-1-18 22891106 PMC3405778

[B8] Appleton C. T. Mcerlain D. D. Pitelka V. Schwartz N. Bernier S. M. Henry J. L. (2007). Forced mobilization accelerates pathogenesis: characterization of a preclinical surgical model of osteoarthritis. *Arthritis Res. Ther.* 9:R13 10.1186/ar2120 PMC1860072 17284317

[B9] Aran S. Daftari Besheli L. Karcaaltincaba M. Gupta R. Flores E. J. Abujudeh H. H. (2014). Applications of dual-energy CT in emergency radiology. *AJR Am. J. Roentgenol.* 202 W314–W324. 10.2214/AJR.13.11682 24660729

[B10] Artaechevarria X. Blanco D. De Biurrun G. Ceresa M. Perez-Martin D. Bastarrika G. (2011). Evaluation of micro-CT for emphysema assessment in mice: comparison with non-radiological techniques. *Eur. Radiol.* 21 954–962. 10.1007/s00330-010-1982-5 20953986

[B11] Artaechevarria X. Blanco D. Perez-Martin D. De Biurrun G. Montuenga L. M. De Torres J. P. (2010). Longitudinal study of a mouse model of chronic pulmonary inflammation using breath hold gated micro-CT. *Eur. Radiol.* 20 2600–2608. 10.1007/s00330-010-1853-0 20574632

[B12] Ashton J. R. Befera N. Clark D. Qi Y. Mao L. Rockman H. A. (2014a). Anatomical and functional imaging of myocardial infarction in mice using micro-CT and eXIA 160 contrast agent. *Contrast Media Mol. Imaging* 9 161–168. 10.1002/cmmi.1557 24523061 PMC4017375

[B13] Ashton J. R. Clark D. P. Moding E. J. Ghaghada K. Kirsch D. G. West J. L. (2014b). Dual-energy micro-CT functional imaging of primary lung cancer in mice using gold and iodine nanoparticle contrast agents: a validation study. *PLoS ONE* 9:e88129 10.1371/journal.pone.0088129 PMC3919743 24520351

[B14] Aviv H. Bartling S. Kieslling F. Margel S. (2009). Radiopaque iodinated copolymeric nanoparticles for X-ray imaging applications. *Biomaterials* 30 5610–5616. 10.1016/j.biomaterials.2009.06.038 19592085

[B15] Badea C. T. Athreya K. K. Espinosa G. Clark D. Ghafoori A. P. Li Y. (2012). Computed tomography imaging of primary lung cancer in mice using a liposomal-iodinated contrast agent. *PLoS ONE* 7:e34496 10.1371/journal.pone.0034496 PMC3317632 22485175

[B16] Badea C. Fubara B. Hedlund L. Johnson G. (2005). 4D micro-CT of the mouse heart. *Mol. Imaging* 4 110–116. 16105509 10.1162/15353500200504187

[B17] Badea C. Hedlund L. W. Johnson G. A. (2004). Micro-CT with respiratory and cardiac gating. *Med. Phys.* 31 3324–3329. 10.1118/1.1812604 15651615 PMC1357396

[B18] Badea C. Schreibmann E. Fox T. (2008a). A registration based approach for 4D cardiac micro-CT using combined prospective and retrospective gating. *Med. Phys.* 35 1170–1179. 10.1118/1.2868778 18491508 PMC2312091

[B19] Badea C. T. Wetzel A. W. Mistry N. Pomerantz S. Nave D. Johnson G. A. (2008b). Left ventricle volume measurements in cardiac micro-CT: the impact of radiation dose and contrast agent. *Comput. Med. Imaging Graph.* 32 239–250. 10.1016/j.compmedimag.2007.12.004 18243656 PMC2276250

[B20] Badea C. T. Ghaghada K. Espinosa G. Strong L. Annapragada A. (2011a). “Multi-modality PET-CT imaging of breast cancer in an animal model using nanoparticle x-ray contrast agent and 18F-FDG,” in *Proceedings of the SPIE 7965 Medical Imaging 2011: Biomedical Applications in Molecular, Structural, and Functional Imaging*, Vol. 796511 (Lake Buena Vista, FL: SPIE Digital Library).

[B21] Badea C. T. Hedlund L. W. Cook J. Berridge B. R. Johnson G. A. (2011b). Micro-CT imaging assessment of dobutamine-induced cardiac stress in rats. *J. Pharmacol. Toxicol. Methods* 63 24–29. 10.1016/j.vascn.2010.04.002 20399875 PMC2916074

[B22] Badea C. T. Johnston S. M. Qi Y. Johnson G. A. (2011c). 4D micro-CT for cardiac and perfusion applications with view under sampling. *Phys. Med. Biol.* 56 3351–3369. 10.1088/0031-9155/56/11/011 21558587 PMC3180888

[B23] Badea C. T. Hedlund L. W. De Lin M. Boslego Mackel J. F. Johnson G. A. (2006). Tumor imaging in small animals with a combined micro-CT/micro-DSA system using iodinated conventional and blood pool contrast agents. *Contrast Media Mol. Imaging* 1 153–164. 10.1002/cmmi.103 17193692

[B24] Badea C. T. Hedlund L. W. Mackel J. F. Mao L. Rockman H. A. Johnson G. A. (2007). Cardiac micro-computed tomography for morphological and functional phenotyping of muscle LIM protein null mice. *Mol. Imaging* 6 261–268. 17711781 PMC2671027

[B25] Bartling S. H. Dinkel J. Stiller W. Grasruck M. Madisch I. Kauczor H. U. (2008). Intrinsic respiratory gating in small-animal CT. *Eur. Radiol.* 18 1375–1384. 10.1007/s00330-008-0903-3 18431578

[B26] Bartling S. H. Stiller W. Grasruck M. Schmidt B. Peschke P. Semmler W. (2007). Retrospective motion gating in small animal CT of mice and rats. *Invest. Radiol.* 42 704–714. 10.1097/RLI.0b013e318070dcad 17984768

[B27] Betzer O. Shwartz A. Motiei M. Kazimirsky G. Gispan I. Damti E. (2014). Nanoparticle-based CT imaging technique for longitudinal and quantitative stem cell tracking within the brain: application in neuropsychiatric disorders. *ACS Nano* 8 9274–9285. 10.1021/nn503131h 25133802

[B28] Bhavane R. Badea C. Ghaghada K. B. Clark D. Vela D. Moturu A. (2013). Dual-energy computed tomography imaging of atherosclerotic plaques in a mouse model using a liposomal-iodine nanoparticle contrast agent. *Circ. Cardiovasc. Imaging* 6 285–294. 10.1161/CIRCIMAGING.112.000119 23349231 PMC3760185

[B29] Boll H. Nittka S. Doyon F. Neumaier M. Marx A. Kramer M. (2011). Micro-CT based experimental liver imaging using a nanoparticulate contrast agent: a longitudinal study in mice. *PLoS ONE* 6:e25692 10.1371/journal.pone.0025692 PMC3184160 21984939

[B30] Bongartz T. Glazebrook K. N. Kavros S. J. Murthy N. S. Merry S. P. Franz W. B. (2015). Dual-energy CT for the diagnosis of gout: an accuracy and diagnostic yield study. *Ann. Rheum. Dis.* 74 1072–1077. 10.1136/annrheumdis-2013-205095 24671771 PMC4431329

[B31] Boone J. M. Velazquez O. Cherry S. R. (2004). Small-animal X-ray dose from micro-CT. *Mol. Imaging* 3 149–158. 10.1162/1535350042380326 15530250

[B32] Bourin M. Jolliet P. Ballereau F. (1997). An overview of the clinical pharmacokinetics of x-ray contrast media. *Clin. Pharmacokinet.* 32 180–193. 10.2165/00003088-199732030-00002 9084958

[B33] Bouxsein M. L. Boyd S. K. Christiansen B. A. Guldberg R. E. Jepsen K. J. Muller R. (2010). Guidelines for assessment of bone microstructure in rodents using micro-computed tomography. *J. Bone Miner. Res.* 25 1468–1486. 10.1002/jbmr.141 20533309

[B34] Carlson S. K. Classic K. L. Bender C. E. Russell S. J. (2007). Small animal absorbed radiation dose from serial micro-computed tomography imaging. *Mol. Imaging Biol.* 9 78–82. 10.1007/s11307-007-0080-9 17285239

[B35] Carril M. Fernandez I. Rodriguez J. Garcia I. Penades S. (2014). Gold-coated iron oxide glyconanoparticles for MRI, CT, and US Multimodal Imaging. *Parti. Part. Syst. Charact.* 31 81–87. 10.1002/ppsc.201300239

[B36] Cervenka L. Mitchell K. D. Navar L. G. (1999). Renal function in mice: effects of volume expansion and angiotensin II. *J. Am. Soc. Nephrol.* 10 2631–2636. 10589704 10.1681/ASN.V10122631

[B37] Chanda N. Kattumuri V. Shukla R. Zambre A. Katti K. Upendran A. (2010). Bombesin functionalized gold nanoparticles show in vitro and in vivo cancer receptor specificity. *Proc. Natl. Acad. Sci. U.S.A.* 107 8760–8765. 10.1073/pnas.1002143107 20410458 PMC2889350

[B38] Chen F. Ehlerding E. B. Cai W. (2014). Theranostic nanoparticles. *J. Nucl. Med.* 55 1919–1922. 10.2967/jnumed.114.146019 25413134 PMC4255955

[B39] Choi E. J. Jin G. Y. Bok S. M. Han Y. M. Lee Y. S. Jung M. J. (2014). Serial micro-CT assessment of the therapeutic effects of rosiglitazone in a bleomycin-induced lung fibrosis mouse model. *Korean J. Radiol.* 15 448–455. 10.3348/kjr.2014.15.4.448 25053904 PMC4105807

[B40] Chouker A. Lizak M. Schimel D. Helmberger T. Ward J. M. Despres D. (2008). Comparison of Fenestra VC Contrast-enhanced computed tomography imaging with gadopentetate dimeglumine and ferucarbotran magnetic resonance imaging for the in vivo evaluation of murine liver damage after ischemia and reperfusion. *Invest. Radiol.* 43 77–91. 10.1097/RLI.0b013e318155aa2e 18197060

[B41] Chow P. L. Rannou F. R. Chatziioannou A. F. (2005). Attenuation correction for small animal PET tomographs. *Phys. Med. Biol.* 50 1837–1850. 10.1088/0031-9155/50/8/014 15815099 PMC1479306

[B42] Clark D. P. Badea C. T. (2014). Spectral diffusion: an algorithm for robust material decomposition of spectral CT data. *Phys. Med. Biol.* 59 6445–6466. 10.1088/0031-9155/59/21/6445 25296173 PMC4210864

[B43] Clark D. Ghaghada K. Moding E. Kirsch D. Badea C. (2013). In vivo characterization of tumor vasculature using iodine and gold nanoparticles and dual energy micro-CT. *Phys. Med. Biol.* 58 1683–1704. 10.1088/0031-9155/58/6/1683 23422321 PMC3746324

[B44] Cole L. E. Vargo-Gogola T. Roeder R. K. (2014). Bisphosphonate-functionalized gold nanoparticles for contrast-enhanced X-ray detection of breast microcalcifications. *Biomaterials* 35 2312–2321. 10.1016/j.biomaterials.2013.11.077 24360718 PMC3956113

[B45] Cormode D. P. Naha P. C. Fayad Z. A. (2014). Nanoparticle contrast agents for computed tomography: a focus on micelles. *Contrast Media Mol. Imaging* 9 37–52. 10.1002/cmmi.1551 24470293 PMC3905628

[B46] Cormode D. P. Roessl E. Thran A. Skajaa T. Gordon R. E. Schlomka J. P. (2010). Atherosclerotic plaque composition: analysis with multicolor CT and targeted gold nanoparticles. *Radiology* 256 774–782. 10.1148/radiol.10092473 20668118 PMC2923725

[B47] Coughlin A. J. Ananta J. S. Deng N. Larina I. V. Decuzzi P. West J. L. (2014). Gadolinium-conjugated gold nanoshells for multimodal diagnostic imaging and photothermal cancer therapy. *Small* 10 556–565. 10.1002/smll.201302217 24115690 PMC3972071

[B48] Cowan C. M. Aghaloo T. Chou Y. F. Walder B. Zhang X. Soo C. (2007). MicroCT evaluation of three-dimensional mineralization in response to BMP-2 doses in vitro and in critical sized rat calvarial defects. *Tissue Eng.* 13 501–512. 10.1089/ten.2006.0141 17319794

[B49] Criscione J. M. Dobrucki L. W. Zhuang Z. W. Papademetris X. Simons M. Sinusas A. J. (2011). Development and application of a multimodal contrast agent for SPECT/CT hybrid imaging. *Bioconjug. Chem.* 22 1784–1792. 10.1021/bc200162r 21851119 PMC3204385

[B50] Curry T. Kopelman R. Shilo M. Popovtzer R. (2014). Multifunctional theranostic gold nanoparticles for targeted CT imaging and photothermal therapy. *Contrast Media Mol. Imaging* 9 53–61. 10.1002/cmmi.1563 24470294

[B51] Danila D. Johnson E. Kee P. (2013). CT imaging of myocardial scars with collagen-targeting gold nanoparticles. *Nanomedicine* 9 1067–1076. 10.1016/j.nano.2013.03.009 23563046

[B52] David V. Laroche N. Boudignon B. Lafage-Proust M. H. Alexandre C. Ruegsegger P. (2003). Noninvasive in vivo monitoring of bone architecture alterations in hindlimb-unloaded female rats using novel three-dimensional microcomputed tomography. *J. Bone Miner. Res.* 18 1622–1631. 10.1359/jbmr.2003.18.9.1622 12968671

[B53] De Langhe E. Vande Velde G. Hostens J. Himmelreich U. Nemery B. Luyten F. P. (2012). Quantification of lung fibrosis and emphysema in mice using automated micro-computed tomography. *PLoS ONE* 7:e43123 10.1371/journal.pone.0043123 PMC3418271 22912805

[B54] Desnoyers L. R. Pai R. Ferrando R. E. Hotzel K. Le T. Ross J. (2008). Targeting FGF19 inhibits tumor growth in colon cancer xenograft and FGF19 transgenic hepatocellular carcinoma models. *Oncogene* 27 85–97. 10.1038/sj.onc.1210623 17599042

[B55] de Vries A. Custers E. Lub J. Van Den Bosch S. Nicolay K. Grull H. (2010). Block-copolymer-stabilized iodinated emulsions for use as CT contrast agents. *Biomaterials* 31 6537–6544. 10.1016/j.biomaterials.2010.04.056 20541800

[B56] Diehl K. H. Hull R. Morton D. Pfister R. Rabemampianina Y. Smith D. (2001). A good practice guide to the administration of substances and removal of blood, including routes and volumes. *J. Appl. Toxicol.* 21 15–23. 10.1002/jat.727 11180276

[B57] Dumas A. Brigitte M. Moreau M. F. Chretien F. Basle M. F. Chappard D. (2009). Bone mass and microarchitecture of irradiated and bone marrow-transplanted mice: influences of the donor strain. *Osteoporos. Int.* 20 435–443. 10.1007/s00198-008-0658-3 18548305

[B58] Eck W. Nicholson A. I. Zentgraf H. Semmler W. Bartling S. (2010). Anti-CD4-targeted gold nanoparticles induce specific contrast enhancement of peripheral lymph nodes in X-ray computed tomography of live mice. *Nano Lett.* 10 2318–2322. 10.1021/nl101019s 20496900

[B59] Ekdawi S. N. Stewart J. M. Dunne M. Stapleton S. Mitsakakis N. Dou Y. N. (2015). Spatial and temporal mapping of heterogeneity in liposome uptake and microvascular distribution in an orthotopic tumor xenograft model. *J. Control. Release* 207 101–111. 10.1016/j.jconrel.2015.04.006 25862513

[B60] Erathodiyil N. Ying J. Y. (2011). Functionalization of inorganic nanoparticles for bioimaging applications. *Acc. Chem. Res.* 44 925–935. 10.1021/ar2000327 21648430

[B61] Feldkamp L. A. Davis L. C. Kress J. W. (1984). Practical cone-beam algorithm. *J. Opt. Soc. Am.* 1 612–619. 10.1364/JOSAA.1.000612

[B62] Feldkamp L. A. Goldstein S. A. Parfitt A. M. Jesion G. Kleerekoper M. (1989). The direct examination of three-dimensional bone architecture in vitro by computed tomography. *J. Bone Miner. Res.* 4 3–11. 10.1002/jbmr.5650040103 2718776

[B63] Feng G. Kong B. Xing J. Chen J. (2014). Enhancing multimodality functional and molecular imaging using glucose-coated gold nanoparticles. *Clin. Radiol.* 69 1105–1111. 10.1016/j.crad.2014.05.112 25023059

[B64] Fiebig T. Boll H. Figueiredo G. Kerl H. U. Nittka S. Groden C. (2012). Three-dimensional in vivo imaging of the murine liver: a micro-computed tomography-based anatomical study. *PLoS ONE* 7:e31179 10.1371/journal.pone.0031179 PMC3280110 22363574

[B65] Ford N. L. Nikolov H. N. Norley C. J. Thornton M. M. Foster P. J. Drangova M. (2005). Prospective respiratory-gated micro-CT of free breathing rodents. *Med. Phys.* 32 2888–2898. 10.1118/1.2013007 16266103

[B66] Ford N. L. Wheatley A. R. Holdsworth D. W. Drangova M. (2007). Optimization of a retrospective technique for respiratory-gated high speed micro-CT of free-breathing rodents. *Phys. Med. Biol.* 52 5749–5769. 10.1088/0031-9155/52/19/002 17881798

[B67] Galperin A. Margel D. Baniel J. Dank G. Biton H. Margel S. (2007). Radiopaque iodinated polymeric nanoparticles for X-ray imaging applications. *Biomaterials* 28 4461–4468. 10.1016/j.biomaterials.2007.06.032 17644171

[B68] Ghaghada K. B. Badea C. T. Karumbaiah L. Fettig N. Bellamkonda R. V. Johnson G. (2011). Evaluation of tumor microenvironment in an animal model using a nanoparticle contrast agent in computed tomography imaging. *Acad. Radiol.* 18 20–30. 10.1016/j.acra.2010.09.003 21145026 PMC3016875

[B69] Ghann W. E. Aras O. Fleiter T. Daniel M. C. (2012). Syntheses and characterization of lisinopril-coated gold nanoparticles as highly stable targeted CT contrast agents in cardiovascular diseases. *Langmuir* 28 10398–10408. 10.1021/la301694q 22702239

[B70] Goertzen A. L. Meadors A. K. Silverman R. W. Cherry S. R. (2002). Simultaneous molecular and anatomical imaging of the mouse in vivo. *Phys. Med. Biol.* 47 4315–4328. 10.1088/0031-9155/47/24/301 12539974

[B71] Graham K. C. Ford N. L. Mackenzie L. T. Postenka C. O. Groom A. C. Macdonald I. C. (2008). Noninvasive quantification of tumor volume in preclinical liver metastasis models using contrast-enhanced x-ray computed tomography. *Invest. Radiol.* 43 92–99. 10.1097/RLI.0b013e31815603d7 18197061

[B72] Guerrero T. Castillo R. Sanders K. Price R. Komaki R. Cody D. (2006). Novel method to calculate pulmonary compliance images in rodents from computed tomography acquired at constant pressures. *Phys. Med. Biol.* 51 1101–1112. 10.1088/0031-9155/51/5/003 16481680

[B73] Guldberg R. E. Lin A. S. Coleman R. Robertson G. Duvall C. (2004). Microcomputed tomography imaging of skeletal development and growth. *Birth Defects Res. C Embryo Today* 72 250–259. 10.1002/bdrc.20016 15495187

[B74] Guo X. Johnston S. M. Qi Y. Johnson G. A. Badea C. T. (2011). 4D micro-CT using fast prospective gating. *Phys. Med. Biol.* 57 257–271. 10.1088/0031-9155/57/1/257 22156062 PMC3388603

[B75] Hainfeld J. F. Dilmanian F. A. Slatkin D. N. Smilowitz H. M. (2008). Radiotherapy enhancement with gold nanoparticles. *J. Pharm. Pharmacol.* 60 977–985. 10.1211/jpp.60.8.0005 18644191

[B76] Hainfeld J. F. Dilmanian F. A. Zhong Z. Slatkin D. N. Kalef-Ezra J. A. Smilowitz H. M. (2010). Gold nanoparticles enhance the radiation therapy of a murine squamous cell carcinoma. *Phys. Med. Biol.* 55 3045–3059. 10.1088/0031-9155/55/11/004 20463371

[B77] Hainfeld J. F. Lin L. Slatkin D. N. Avraham Dilmanian F. Vadas T. M. Smilowitz H. M. (2014). Gold nanoparticle hyperthermia reduces radiotherapy dose. *Nanomedicine* 10 1609–1617. 10.1016/j.nano.2014.05.006 24990355 PMC4253648

[B78] Hainfeld J. F. O’Connor M. J. Dilmanian F. A. Slatkin D. N. Adams D. J. Smilowitz H. M. (2011). Micro-CT enables microlocalisation and quantification of Her2-targeted gold nanoparticles within tumour regions. *Br. J. Radiol.* 84 526–533. 10.1259/bjr/42612922 21081567 PMC3473629

[B79] Hainfeld J. F. Slatkin D. N. Focella T. M. Smilowitz H. M. (2006). Gold nanoparticles: a new X-ray contrast agent. *Br. J. Radiol.* 79 248–253. 10.1259/bjr/13169882 16498039

[B80] Hainfeld J. F. Slatkin D. N. Smilowitz H. M. (2004). The use of gold nanoparticles to enhance radiotherapy in mice. *Phys. Med. Biol.* 49 N309–N315. 10.1088/0031-9155/49/18/N03 15509078

[B81] Hainfeld J. F. Smilowitz H. M. O’Connor M. J. Dilmanian F. A. Slatkin D. N. (2013). Gold nanoparticle imaging and radiotherapy of brain tumors in mice. *Nanomedicine* (*Lond*.) 8 1601–1609. 10.2217/nnm.12.165 23265347 PMC3657324

[B82] Hallouard F. Anton N. Choquet P. Constantinesco A. Vandamme T. (2010). Iodinated blood pool contrast media for preclinical X-ray imaging applications–a review. *Biomaterials* 31 6249–6268. 10.1016/j.biomaterials.2010.04.066 20510444

[B83] Hallouard F. Briancon S. Anton N. Li X. Vandamme T. Fessi H. (2013). Iodinated nano-emulsions as contrast agents for preclinical X-ray imaging: impact of the free surfactants on the pharmacokinetics. *Eur. J. Pharm. Biopharm.* 83 54–62. 10.1016/j.ejpb.2012.09.003 23010566

[B84] Hedlund L. W. Johnson G. A. (2002). Mechanical ventilation for imaging the small animal lung. *ILAR J.* 43 159–174. 10.1093/ilar.43.3.159 12105383

[B85] Ho S. T. Hutmacher D. W. (2006). A comparison of micro CT with other techniques used in the characterization of scaffolds. *Biomaterials* 27 1362–1376. 10.1016/j.biomaterials.2005.08.035 16174523

[B86] Hori Y. Takasuka N. Mutoh M. Kitahashi T. Kojima S. Imaida K. (2008). Periodic analysis of urethane-induced pulmonary tumors in living A/J mice by respiration-gated X-ray microcomputed tomography. *Cancer Sci.* 99 1774–1777. 10.1111/j.1349-7006.2008.00889.x 18616525 PMC11159843

[B87] Hsu J. T. Chen Y. J. Ho J. T. Huang H. L. Wang S. P. Cheng F. C. (2014). A comparison of micro-CT and dental CT in assessing cortical bone morphology and trabecular bone microarchitecture. *PLoS ONE* 9:e107545 10.1371/journal.pone.0107545 PMC4166457 25226587

[B88] Huang P. Bao L. Zhang C. Lin J. Luo T. Yang D. (2011). Folic acid-conjugated silica-modified gold nanorods for X-ray/CT imaging-guided dual-mode radiation and photo-thermal therapy. *Biomaterials* 32 9796–9809. 10.1016/j.biomaterials.2011.08.086 21917309

[B89] Hupfer M. Nowak T. Brauweiler R. Eisa F. Kalender W. A. (2012). Spectral optimization for micro-CT. *Med. Phys.* 39 3229–3239. 10.1118/1.4718575 22755706

[B90] Hwang A. B. Hasegawa B. H. (2005). Attenuation correction for small animal SPECT imaging using x-ray CT data. *Med. Phys.* 32 2799–2804. 10.1118/1.1984347 16266094

[B91] Jakhmola A. Anton N. Vandamme T. F. (2012). Inorganic nanoparticles based contrast agents for X-ray computed tomography. *Adv. Healthc. Mater* 1 413–431. 10.1002/adhm.201200032 23184772

[B92] Jepperson M. A. Cernigliaro J. G. Sella D. Ibrahim E. Thiel D. D. Leng S. (2013). Dual-energy CT for the evaluation of urinary calculi: image interpretation, pitfalls and stone mimics. *Clin. Radiol.* 68 e707–e714. 10.1016/j.crad.2013.07.012 23988091

[B93] Jeremic B. Aguerri A. R. Filipovic N. (2013). Radiosensitization by gold nanoparticles. *Clin. Trans. Oncol.* 15 593–601. 10.1007/s12094-013-1003-7 23359187

[B94] Jiang S. D. Jiang L. S. Dai L. Y. (2006). Spinal cord injury causes more damage to bone mass, bone structure, biomechanical properties and bone metabolism than sciatic neurectomy in young rats. *Osteoporos. Int.* 17 1552–1561. 10.1007/s00198-006-0165-3 16874443

[B95] Johnson K. A. (2007). Imaging techniques for small animal imaging models of pulmonary disease: Micro-CT. *Toxicol. Pathol.* 35 59–64. 10.1080/01926230601184262 17325973 PMC2094132

[B96] Johnston S. M. Perez B. A. Kirsch D. G. Badea C. T. (2010). Phase-selective image reconstruction of the lungs in small animals using Micro-CT. *Proc. SPIE Int. Soc. Opt. Eng.* 7622 76223G.1–76223G.9. 10.1117/12.844359 PMC3018736 21243034

[B97] Jokerst J. V. Lobovkina T. Zare R. N. Gambhir S. S. (2011). Nanoparticle PEGylation for imaging and therapy. *Nanomedicine* (*Lond*.) 6 715–728. 10.2217/nnm.11.19 21718180 PMC3217316

[B98] Key J. Leary J. F. (2014). Nanoparticles for multimodal in vivo imaging in nanomedicine. *Int. J. Nanomed.* 9 711–726. 10.2147/IJN.S53717 PMC3915020 24511229

[B99] Kiessling F. Greschus S. Lichy M. P. Bock M. Fink C. Vosseler S. (2004). Volumetric computed tomography (VCT): a new technology for noninvasive, high-resolution monitoring of tumor angiogenesis. *Nat. Med.* 10 1133–1138. 10.1038/nm1101 15361864

[B100] Kim H. W. Cai Q. Y. Jun H. Y. Chon K. S. Park S. H. Byun S. J. (2008). Micro-CT imaging with a hepatocyte-selective contrast agent for detecting liver metastasis in living mice. *Acad. Radiol.* 15 1282–1290. 10.1016/j.acra.2008.03.021 18790400

[B101] Kindlmann G. L. Weinstein D. M. Jones G. M. Johnson C. R. Capecchi M. R. Keller C. (2005). Practical vessel imaging by computed tomography in live transgenic mouse models for human tumors. *Mol. Imaging* 4 417–424. 16285903 10.2310/7290.2005.05166

[B102] Kinney J. H. Lane N. E. Haupt D. L. (1995). In vivo, three-dimensional microscopy of trabecular bone. *J. Bone Miner. Res.* 10 264–270. 10.1002/jbmr.5650100213 7754806

[B103] Kircher M. F. Willmann J. K. (2012). Molecular body imaging: MR imaging, CT, and US. part I. principles. *Radiology* 263 633–643. 10.1148/radiol.12102394 22623690 PMC3359513

[B104] Kirsch D. G. Grimm J. Guimaraes A. R. Wojtkiewicz G. R. Perez B. A. Santiago P. M. (2010). Imaging primary lung cancers in mice to study radiation biology. *Int. J. Radiat. Oncol. Biol. Phys.* 76 973–977. 10.1016/j.ijrobp.2009.11.038 20206017 PMC2847457

[B105] Krause W. Leike J. Sachse A. Schuhmann-Giampieri G. (1993). Characterization of iopromide liposomes. *Invest. Radiol.* 28 1028–1032. 10.1097/00004424-199311000-00011 8276573

[B106] Kuntz J. Dinkel J. Zwick S. Bauerle T. Grasruck M. Kiessling F. (2010). Fully automated intrinsic respiratory and cardiac gating for small animal CT. *Phys. Med. Biol.* 55 2069–2085. 10.1088/0031-9155/55/7/018 20299735

[B107] Laib A. Barou O. Vico L. Lafage-Proust M. H. Alexandre C. Rugsegger P. (2000). 3D micro-computed tomography of trabecular and cortical bone architecture with application to a rat model of immobilisation osteoporosis. *Med. Biol. Eng. Comput.* 38 326–332. 10.1007/BF02347054 10912350

[B108] Laib A. Kumer J. L. Majumdar S. Lane N. E. (2001). The temporal changes of trabecular architecture in ovariectomized rats assessed by MicroCT. *Osteoporos. Int.* 12 936–941. 10.1007/s001980170022 11804020

[B109] Langheinrich A. C. Ritman E. L. (2006). Quantitative imaging of microvascular permeability in a rat model of lipopolysaccharide-induced sepsis: evaluation using cryostatic micro-computed tomography. *Invest. Radiol.* 41 645–650. 10.1097/01.rli.0000227494.17444.64 16829748

[B110] Lee C. L. Min H. Befera N. Clark D. Qi Y. Das S. (2014). Assessing cardiac injury in mice with dual energy-microCT, 4D-microCT, and microSPECT imaging after partial heart irradiation. *Int. J. Radiat. Oncol. Biol. Phys.* 88 686–693. 10.1016/j.ijrobp.2013.11.238 24521682 PMC3985387

[B111] Lee S. W. Padmanabhan P. Ray P. Gambhir S. S. Doyle T. Contag C. (2009). Stem cell-mediated accelerated bone healing observed with in vivo molecular and small animal imaging technologies in a model of skeletal injury. *J. Orthop. Res.* 27 295–302. 10.1002/jor.20736 18752273 PMC4154812

[B112] Li M. Jirapatnakul A. Biancardi A. Riccio M. L. Weiss R. S. Reeves A. P. (2013a). Growth pattern analysis of murine lung neoplasms by advanced semi-automated quantification of micro-CT images. *PLoS ONE* 8:e83806 10.1371/journal.pone.0083806 PMC3871568 24376755

[B113] Li X. Anton N. Zuber G. Zhao M. Messaddeq N. Hallouard F. (2013b). Iodinated alpha-tocopherol nano-emulsions as non-toxic contrast agents for preclinical X-ray imaging. *Biomaterials* 34 481–491. 10.1016/j.biomaterials.2012.09.026 23083930

[B114] Li R. Stewart D. J. Von Schroeder H. P. Mackinnon E. S. Schemitsch E. H. (2009). Effect of cell-based VEGF gene therapy on healing of a segmental bone defect. *J. Orthop. Res.* 27 8–14. 10.1002/jor.20658 18634016

[B115] Li X. Anton N. Zuber G. Vandamme T. (2014). Contrast agents for preclinical targeted X-ray imaging. *Adv. Drug Deliv. Rev.* 76 116–133. 10.1016/j.addr.2014.07.013 25086373

[B116] Li X. F. Zanzonico P. Ling C. C. O’Donoghue J. (2006). Visualization of experimental lung and bone metastases in live nude mice by X-ray micro-computed tomography. *Technol. Cancer Res. Treat.* 5 147–155. 16551134

[B117] Li X. M. Wang L. Fan Y. B. Feng Q. L. Cui F. Z. (2012). Biocompatibility and toxicity of nanoparticles and Nanotubes. *J. Nanomater.* 2012:548389 10.1155/2012/591278

[B118] Liang H. Yang Y. Yang K. Wu Y. Boone J. M. Cherry S. R. (2007). A microPET/CT system for in vivo small animal imaging. *Phys. Med. Biol.* 52 3881–3894. 10.1088/0031-9155/52/13/015 17664583

[B119] Lin C. Y. Schek R. M. Mistry A. S. Shi X. Mikos A. G. Krebsbach P. H. (2005). Functional bone engineering using ex vivo gene therapy and topology-optimized, biodegradable polymer composite scaffolds. *Tissue Eng.* 11 1589–1598. 10.1089/ten.2005.11.1589 16259612

[B120] Lin M. D. Marshall C. T. Qi Y. Badea C. Piantadosi C. Johnson G. A. (2009). Quantitative blood flow measurements in the small animal cardi-pulmonary system using x-ray digital subtraction angiography. *Med. Phys.* 36 5347–5358. 10.1118/1.3231823 19994543 PMC2780468

[B121] Liu Y. Ashton J. R. Moding E. J. Yuan H. Register J. K. Fales A. M. (2015). A Plasmonic gold nanostar theranostic probe for in vivo tumor imaging and photothermal therapy. *Theranostics* 5 946–960. 10.7150/thno.11974 26155311 PMC4493533

[B122] Lu Z. R. (2014). Theranostics: fusion of therapeutics and diagnostics. *Pharm. Res.* 31 1355–1357. 10.1007/s11095-014-1343-1 24623483

[B123] Lusic H. Grinstaff M. W. (2013). X-Ray computed tomography contrast agents. *Chem. Rev.* 113 1641–1666. 10.1021/cr200358s 23210836 PMC3878741

[B124] Maeda H. (2001). The enhanced permeability and retention (EPR) effect in tumor vasculature: the key role of tumor-selective macromolecular drug targeting. *Adv. Enzyme Regul.* 41 189–207. 10.1016/S0065-2571(00)00013-3 11384745

[B125] Maeda H. Wu J. Sawa T. Matsumura Y. Hori K. (2000). Tumor vascular permeability and the EPR effect in macromolecular therapeutics: a review. *J. Control. Release* 65 271–284. 10.1016/S0168-3659(99)00248-5 10699287

[B126] Marin D. Boll D. T. Mileto A. Nelson R. C. (2014). State of the art: dual-energy CT of the abdomen. *Radiology* 271 327–342. 10.1148/radiol.14131480 24761954

[B127] McErlain D. D. Appleton C. T. Litchfield R. B. Pitelka V. Henry J. L. Bernier S. M. (2008). Study of subchondral bone adaptations in a rodent surgical model of OA using in vivo micro-computed tomography. *Osteoarthritis Cartilage* 16 458–469. 10.1016/j.joca.2007.08.006 17900933 PMC5130342

[B128] Mileto A. Marin D. Nelson R. C. Ascenti G. Boll D. T. (2014). Dual energy MDCT assessment of renal lesions: an overview. *Eur. Radiol.* 24 353–362. 10.1007/s00330-013-3030-8 24092045

[B129] Moding E. J. Clark D. P. Qi Y. Li Y. Ma Y. Ghaghada K. (2013). Dual-energy micro-computed tomography imaging of radiation-induced vascular changes in primary mouse sarcomas. *Int. J. Radiat. Oncol. Biol. Phys.* 85 1353–1359. 10.1016/j.ijrobp.2012.09.027 23122984 PMC3625949

[B130] Moghimi S. M. Hunter A. C. Murray J. C. (2001). Long-circulating and target-specific nanoparticles: theory to practice. *Pharmacol. Rev.* 53 283–318. 11356986

[B131] Montet X. Pastor C. M. Vallee J. P. Becker C. D. Geissbuhler A. Morel D. R. (2007). Improved visualization of vessels and hepatic tumors by micro-computed tomography (CT) using iodinated liposomes. *Invest. Radiol.* 42 652–658. 10.1097/RLI.0b013e31805f445b 17700281

[B132] Mukundan S. Ghaghada K. Badea C. Hedlund L. Johnson G. Provenzale J. (2006). A nanoscale, liposomal contrast agent for preclincal microct imaging of the mouse. *AJR Am J Roentgenol.* 186 300–307. 16423931 10.2214/AJR.05.0523

[B133] Mulder W. J. Strijkers G. J. Van Tilborg G. A. Griffioen A. W. Nicolay K. (2006). Lipid-based nanoparticles for contrast-enhanced MRI and molecular imaging. *NMR Biomed.* 19 142–164. 10.1002/nbm.1011 16450332

[B134] Munoz-Barrutia A. Ceresa M. Artaechevarria X. Montuenga L. M. Ortiz-De-Solorzano C. (2012). Quantification of lung damage in an elastase-induced mouse model of emphysema. *Int. J. Biomed. Imaging* 2012 734734 10.1155/2012/734734 PMC3503307 23197972

[B135] Nahrendorf M. Badea C. Hedlund L. W. Figueiredo J. L. Sosnovik D. E. Johnson G. A. (2007). High-resolution imaging of murine myocardial infarction with delayed-enhancement cine micro-CT. *Am. J. Physiol. Heart Circ. Physiol.* 292 H3172–H3178. 10.1152/ajpheart.01307.2006 17322414 PMC2680216

[B136] Namasivayam S. Kalra M. K. Torres W. E. Small W. C. (2006). Adverse reactions to intravenous iodinated contrast media: an update. *Curr. Probl. Diagn. Radiol.* 35 164–169. 10.1067/j.cpradiol.2006.04.001 16814003

[B137] Namati E. Chon D. Thiesse J. Hoffman E. A. De Ryk J. Ross A. (2006). In vivo micro-CT lung imaging via a computer-controlled intermittent iso-pressure breath hold (IIBH) technique. *Phys. Med. Biol.* 51 6061–6075. 10.1088/0031-9155/51/23/008 17110770

[B138] Namati E. Thiesse J. Sieren J. C. Ross A. Hoffman E. A. Mclennan G. (2010). Longitudinal assessment of lung cancer progression in the mouse using in vivo micro-CT imaging. *Med. Phys.* 37 4793–4805. 10.1118/1.3476454 20964199 PMC2937054

[B139] Ohana M. Jeung M. Y. Labani A. El Ghannudi S. Roy C. (2014). Thoracic dual energy CT: acquisition protocols, current applications and future developments. *Diagn. Interv. Imaging* 95 1017–1026. 10.1016/j.diii.2014.01.001 24780370

[B140] Pan D. Roessl E. Schlomka J. P. Caruthers S. D. Senpan A. Scott M. J. (2010). Computed tomography in color: nanok-enhanced spectral CT molecular imaging. *Angewandte Chem. Int. Ed.* 49 9635–9639. 10.1002/anie.201005657 PMC3096064 21077082

[B141] Pan D. Schirra C. O. Senpan A. Schmieder A. H. Stacy A. J. Roessl E. (2012). An early investigation of ytterbium nanocolloids for selective and quantitative “multicolor” spectral CT imaging. *ACS Nano* 6 3364–3370. 10.1021/nn300392x 22385324 PMC3529639

[B142] Park J. Park J. Ju E. J. Park S. S. Choi J. Lee J. H. (2015). Multifunctional hollow gold nanoparticles designed for triple combination therapy and CT imaging. *J. Control. Release* 207 77–85. 10.1016/j.jconrel.2015.04.007 25863273

[B143] Parkins C. S. Fowler J. F. Maughan R. L. Roper M. J. (1985). Repair in mouse lung for up to 20 fractions of X rays or neutrons. *Br. J. Radiol.* 58 225–241. 10.1259/0007-1285-58-687-225 4063664

[B144] Paul J. Vogl T. J. Mbalisike E. C. (2014). Oncological applications of dual-energy computed tomography imaging. *J. Comput. Assist. Tomogr.* 38 834–842. 10.1097/RCT.0000000000000133 25032806

[B145] Perera V. S. Hao J. Gao M. Gough M. Zavalij P. Y. Flask C. (2011). Nanoparticles of the novel coordination polymer KBi(H2O)2[Fe(CN)6].H2O as a potential contrast agent for computed tomography. *Inorg Chem.* 50 7910–7912. 10.1021/ic200587s 21797245

[B146] Perez B. Ghafoori A. Johnston S. Jeffords L. Kim Y. Badea C. (2009). Dissecting the mechanism of tumor response to radiation therapy with primary lung cancers in mice. *Int. J. Radiat. Oncol. Biol. Phys.* 75 S537–S537. 10.1016/j.ijrobp.2009.07.1227

[B147] Perez B. A. Ghafoori A. P. Lee C. L. Johnston S. M. Li Y. Moroshek J. G. (2013). Assessing the radiation response of lung cancer with different gene mutations using genetically engineered mice. *Front. Oncol.* 3:72 10.3389/fonc.2013.00072 PMC3613757 23565506

[B148] Petersein J. Franke B. Fouillet X. Hamm B. (1999). Evaluation of liposomal contrast agents for liver CT in healthy rabbits. *Invest. Radiol.* 34 401–409. 10.1097/00004424-199906000-00003 10353032

[B149] Postnov A. A. Meurrens K. Weiler H. Van Dyck D. Xu H. Terpstra P. (2005). In vivo assessment of emphysema in mice by high resolution X-ray microtomography. *J. Microsc.* 220 70–75. 10.1111/j.1365-2818.2005.01510.x 16269065

[B150] Rabin O. Manuel Perez J. Grimm J. Wojtkiewicz G. Weissleder R. (2006). An X-ray computed tomography imaging agent based on long-circulating bismuth sulphide nanoparticles. *Nat. Mater.* 5 118–122. 10.1038/nmat1571 16444262

[B151] Reuveni T. Motiei M. Romman Z. Popovtzer A. Popovtzer R. (2011). Targeted gold nanoparticles enable molecular CT imaging of cancer: an in vivo study. *Int. J. Nanomed.* 6 2859–2864. 10.2147/IJN.S25446 PMC3224712 22131831

[B152] Ritman E. L. (2004). Micro-computed tomography-current status and developments. *Annu. Rev. Biomed. Eng.* 6 185–208. 10.1146/annurev.bioeng.6.040803.140130 15255767

[B153] Roa W. Xiong Y. P. Chen J. Yang X. Y. Song K. Yang X. H. (2012). Pharmacokinetic and toxicological evaluation of multi-functional thiol-6-fluoro-6-deoxy-D-glucose gold nanoparticles in vivo. *Nanotechnology* 23:375101 10.1088/0957-4484/23/37/375101 22922305

[B154] Roessl E. Cormode D. Brendel B. Engel K. J. Martens G. Thran A. (2011). Preclinical spectral computed tomography of gold nano-particles. *Nucl. Instrum. Methods Phys. Res. A Accelerators Spectrometers Detectors Assoc. Equipm.* 648 S259–S264. 10.1016/j.nima.2010.11.072

[B155] Rothe J. H. Rudolph I. Rohwer N. Kupitz D. Gregor-Mamoudou B. Derlin T. (2015). Time course of contrast enhancement by micro-CT with dedicated contrast agents in normal mice and mice with hepatocellular carcinoma: comparison of one iodinated and two nanoparticle-based agents. *Acad. Radiol.* 22 169–178. 10.1016/j.acra.2014.07.022 25282584

[B156] Rudyanto R. D. Bastarrika G. De Biurrun G. Agorreta J. Montuenga L. M. Ortiz-De-Solorzano C. (2013). Individual nodule tracking in micro-CT images of a longitudinal lung cancer mouse model. *Med. Image Anal.* 17 1095–1105. 10.1016/j.media.2013.07.002 23920346

[B157] Ryu J. H. Lee S. Son S. Kim S. H. Leary J. F. Choi K. (2014). Theranostic nanoparticles for future personalized medicine. *J. Control. Release* 190 477–484. 10.1016/j.jconrel.2014.04.027 24780269

[B158] Sagar N. Pandey A. K. Gurbani D. Khan K. Singh D. Chaudhari B. P. (2013). In-vivo efficacy of compliant 3D nano-composite in critical-size bone defect repair: a six month preclinical study in rabbit. *PLoS ONE* 8:e77578 10.1371/journal.pone.0077578 PMC3799616 24204879

[B159] Saito S. Murase K. (2012). Detection and early phase assessment of radiation-induced lung injury in mice using micro-CT. *PLoS ONE* 7:e45960 10.1371/journal.pone.0045960 PMC3454347 23029340

[B160] Samei E. Saunders R. S. Badea C. T. Ghaghada K. B. Hedlund L. W. Qi Y. (2009). Micro-CT imaging of breast tumors in rodents using a liposomal, nanoparticle contrast agent. *Int. J. Nanomed.* 4 277–282. 10.2147/IJN.S7881 PMC2789439 20011244

[B161] Sasaki M. Chubachi S. Kameyama N. Sato M. Haraguchi M. Miyazaki M. (2015). Evaluation of cigarette smoke-induced emphysema in mice using quantitative micro computed tomography. *Am. J. Physiol. Lung Cell Mol. Physiol.* 308 L1039–L1045. 10.1152/ajplung.00366.2014 25820526

[B162] Sawall S. Kuntz J. Socher M. Knaup M. Hess A. Bartling S. (2012). Imaging of cardiac perfusion of free-breathing small animals using dynamic phase-correlated micro-CT. *Med. Phys.* 39 7499–7506. 10.1118/1.4762685 23231299

[B163] Schambach S. J. Bag S. Groden C. Schilling L. Brockmann M. A. (2010). Vascular imaging in small rodents using micro-CT. *Methods* 50 26–35. 10.1016/j.ymeth.2009.09.003 19772922

[B164] Schirra C. O. Brendel B. Anastasio M. A. Roessl E. (2014). Spectral CT: a technology primer for contrast agent development. *Contrast Media Mol. Imaging* 9 62–70. 10.1002/cmmi.1573 24470295

[B165] Schirra C. O. Pan D. P. J. Roessl E. Senpan A. Schmirder A. H. Scott M. (2012). Optimized ruptured plaque detection with ytterbium nanocolloids and spectral CT. *Circulation* 126 A13493.

[B166] Scotton C. J. Hayes B. Alexander R. Datta A. Forty E. J. Mercer P. F. (2013). Ex vivo micro-computed tomography analysis of bleomycin-induced lung fibrosis for preclinical drug evaluation. *Eur. Respir. J.* 42 1633–1645. 10.1183/09031936.00182412 23520313

[B167] Sheikh A. Y. Van Der Bogt K. E. A. Doyle T. C. Sheikh M. K. Ransohoff K. J. Ali Z. A. (2010). Micro-CT for Characterization of Murine CV Disease Models. *Jacc-Cardiovasc. Imaging* 3 783–785. 10.1016/j.jcmg.2010.01.012 20633858 PMC2952324

[B168] Shofer S. Badea C. Auerbach S. Schwartz D. A. Johnson G. A. (2007). A micro-computed tomography-based method for the measurement of pulmonary compliance in healthy and bleomycin-exposed mice. *Exp. Lung. Res.* 33 169–183. 10.1080/01902140701364458 17558678 PMC2677683

[B169] Shofer S. Badea C. Qi Y. Potts E. Foster W. M. Johnson G. A. (2008). A micro-CT analysis of murine lung recruitment in bleomycin-induced lung injury. *J. Appl. Physiol.* 105 669–677. 10.1152/japplphysiol.00980.2007 18566189 PMC2519942

[B170] Song J. Liu Q. H. Johnson G. A. Badea C. T. (2007). Sparseness prior based iterative image reconstruction for retrospectively gated cardiac micro-CT. *Med. Phys.* 34 4476–4483. 10.1118/1.2795830 18072512 PMC2366112

[B171] Swy E. R. Schwartz-Duval A. S. Shuboni D. D. Latourette M. T. Mallet C. L. Parys M. (2014). Dual-modality, fluorescent, PLGA encapsulated bismuth nanoparticles for molecular and cellular fluorescence imaging and computed tomography. *Nanoscale* 6 13104–13112. 10.1039/c4nr01405g 25248645 PMC4362618

[B172] Taguchi K. Iwanczyk J. S. (2013). Vision 20/20: single photon counting x-ray detectors in medical imaging. *Med. Phys.* 40:100901 10.1118/1.4820371 PMC3786515 24089889

[B173] Tepel M. Aspelin P. Lameire N. (2006). Contrast-induced nephropathy: a clinical and evidence-based approach. *Circulation* 113 1799–1806. 10.1161/CIRCULATIONAHA.105.595090 16606801

[B174] Torchilin V. P. Frank-Kamenetsky M. D. Wolf G. L. (1999). CT visualization of blood pool in rats by using long-circulating, iodine-containing micelles. *Acad. Radiol.* 6 61–65. 10.1016/S1076-6332(99)80063-4 9891154

[B175] Trubetskoy V. S. Gazelle G. S. Wolf G. L. Torchilin V. P. (1997). Block-copolymer of polyethylene glycol and polylysine as a carrier of organic iodine: design of long-circulating particulate contrast medium for X-ray computed tomography. *J. Drug Target.* 4 381–388. 10.3109/10611869709017895 9239578

[B176] Ueno T. Imaida K. Yoshimoto M. Hayakawa T. Takahashi M. Imai T. (2012). Non-invasive X-ray micro-computed tomographic evaluation of indomethacin on urethane-induced lung carcinogenesis in mice. *Anticancer Res.* 32 4773–4780. 23155242

[B177] Umoh J. U. Sampaio A. V. Welch I. Pitelka V. Goldberg H. A. Underhill T. M. (2009). In vivo micro-CT analysis of bone remodeling in a rat calvarial defect model. *Phys. Med. Biol.* 54 2147–2161. 10.1088/0031-9155/54/7/020 19287088

[B178] Vandeghinste B. Trachet B. Renard M. Casteleyn C. Staelens S. Loeys B. (2011). Replacing vascular corrosion casting by in vivo micro-CT imaging for building 3D cardiovascular models in mice. *Mol. Imaging Biol.* 13 78–86. 10.1007/s11307-010-0335-8 20449667

[B179] Varenika V. Fu Y. Maher J. J. Gao D. Kakar S. Cabarrus M. C. (2013). Hepatic fibrosis: evaluation with semiquantitative contrast-enhanced CT. *Radiology* 266 151–158. 10.1148/radiol.12112452 23169796 PMC3528968

[B180] Voelker M. T. Fichtner F. Kasper M. Kamprad M. Sack U. Kaisers U. X. (2014). Characterization of a double-hit murine model of acute respiratory distress syndrome. *Clin. Exp. Pharmacol. Physiol.* 41 844–853. 10.1111/1440-1681.12283 25115497

[B181] Waarsing J. H. Day J. S. Weinans H. (2005). Longitudinal micro-CT scans to evaluate bone architecture. *J Musculoskelet. Neuronal Interact.* 5 310–312. 16340117

[B182] Wang C. L. Cohan R. H. Ellis J. H. Adusumilli S. Dunnick N. R. (2007). Frequency, management, and outcome of extravasation of nonionic iodinated contrast medium in 69,657 intravenous injections. *Radiology* 243 80–87. 10.1148/radiol.2431060554 17392249

[B183] Wang H. Zheng L. Peng C. Shen M. Shi X. Zhang G. (2013). Folic acid-modified dendrimer-entrapped gold nanoparticles as nanoprobes for targeted CT imaging of human lung adencarcinoma. *Biomaterials* 34 470–480. 10.1016/j.biomaterials.2012.09.054 23088841

[B184] Wetzel A. W. Badea C. T. Pomerantz S. M. Mistry N. Nave D. Johnson G. A. (2007). “Measurement and modeling of 4D live mouse heart volumes from CT time series,” in *Proceedings of the SPIE 6491* Vol. 6491 *Videometrics IX*, eds Beraldin J.-A. Remondino F. Shortis M. R. San Jose, CA, 64910J-1.

[B185] Winter P. M. Shukla H. P. Caruthers S. D. Scott M. J. Fuhrhop R. W. Robertson J. D. (2005). Molecular imaging of human thrombus with computed tomography. *Acad. Radiol.* 12(Suppl. 1), S9–S13. 10.1016/j.acra.2005.02.016 16106538

[B186] Wolfe T. Chatterjee D. Lee J. Grant J. D. Bhattarai S. Tailor R. (2015). Targeted gold nanoparticles enhance sensitization of prostate tumors to megavoltage radiation therapy in vivo. *Nanomedicine* 11 1277–1283. 10.1016/j.nano.2014.12.016 25652893 PMC4476911

[B187] Wyss C. Schaefer S. C. Juillerat-Jeanneret L. Lagopoulos L. Lehr H. A. Becker C. D. (2009). Molecular imaging by micro-CT: specific E-selectin imaging. *Eur. Radiol.* 19 2487–2494. 10.1007/s00330-009-1434-2 19440717

[B188] Xue S. H. Wang Y. Wang M. X. Zhang L. Du X. X. Gu H. C. (2014). Iodinated oil-loaded, fluorescent mesoporous silica-coated iron oxide nanoparticles for magnetic resonance imaging/computed tomography/fluorescence trimodal imaging. *Int. J. Nanomed.* 9 2527–2538. 10.2147/IJN.S59754 PMC4039419 24904212

[B189] Yao W. Hadi T. Jiang Y. Lotz J. Wronski T. J. Lane N. E. (2005). Basic fibroblast growth factor improves trabecular bone connectivity and bone strength in the lumbar vertebral body of osteopenic rats. *Osteoporos. Int.* 16 1939–1947. 10.1007/s00198-005-1969-2 16086094 PMC6897353

[B190] Yu S. B. Watson A. D. (1999). Metal-Based X-ray contrast media. *Chem. Rev.* 99 2353–2378. 10.1021/cr980441p 11749484

[B191] Zheng J. Jaffray D. Allen C. (2009). Quantitative CT imaging of the spatial and temporal distribution of liposomes in a rabbit tumor model. *Mol. Pharm.* 6 571–580. 10.1021/mp800234r 19298061

[B192] Zheng J. Perkins G. Kirilova A. Allen C. Jaffray D. A. (2006). Multimodal contrast agent for combined computed tomography and magnetic resonance imaging applications. *Invest. Radiol.* 41 339–348. 10.1097/01.rli.0000186568.50265.64 16481918

[B193] Zhou Y. Chen H. Ambalavanan N. Liu G. Antony V. B. Ding Q. (2015). Noninvasive imaging of experimental lung fibrosis. *Am. J. Respir. Cell Mol. Biol.* 53 8–13. 10.1165/rcmb.2015-0032TR 25679265 PMC4566116

[B194] Zhu J. Y. Zheng L. F. Wen S. H. Tang Y. Q. Shen M. W. Zhang G. X. (2014). Targeted cancer theranostics using alpha-tocopheryl succinate-conjugated multifunctional dendrimer-entrapped gold nanoparticles. *Biomaterials* 35 7635–7646. 10.1016/j.biomaterials.2014.05.046 24927683

